# Factors of pet ownership associated with human health: A representative cross-sectional survey of Switzerland

**DOI:** 10.1371/journal.pone.0352778

**Published:** 2026-07-29

**Authors:** Rahel Marti, Karin Hediger, Elena Pauli, Jan Hattendorf

**Affiliations:** 1 University of Basel, Faculty of Psychology, Basel, Switzerland; 2 Swiss Tropical and Public Health Institute, Department of Epidemiology and Public Health, Allschwil, Switzerland; 3 University of Lucerne, Faculty of Behavioural Sciences and Psychology, Lucerne, Switzerland; 4 Open University, Faculty of Psychology, Heerlen, Netherlands; UFSJ: Universidade Federal de Sao Joao del-Rei, BRAZIL

## Abstract

**Background:**

Studies have discussed the relation between pet ownership and several health outcomes, but the evidence remains inconclusive. It is suggested that factors related to the owner, the animal, and the human–animal relationship might explain general associations in findings. This study investigates differences in health, social support, life satisfaction, loneliness, and psychological well-being by pet ownership and regular animal contact status. It explores whether these outcomes are associated with attachment to the animal, perceived socio-emotional support from the animal, the animal species, and the amount of animal contact and how these variables relate to each other.

**Method:**

We investigated data from a representative survey of 2,328 Swiss participants. We used health behaviour, health status, healthcare use, social support, life satisfaction, loneliness, and psychological well-being to conduct regression analyses, with pet ownership, human–animal contact, attachment to the animal, socio-emotional support from the animal, and the animal species as the predictors, while controlling for socio-demographic variables.

**Results:**

Pet owners reported a slightly lower health status and a slightly higher frequency of unhealthy behaviour. We found no difference in healthcare use, loneliness, life satisfaction, psychological well-being, or the amount of human social support between pet owners and non-pet owners. People with regular animal contact did not differ in health status, health behaviour, healthcare use, loneliness, life satisfaction, psychological well-being, or human social support compared to people with no regular animal contact. Attachment to the animal and the animal species were not related to health, loneliness, life satisfaction, and psychological well-being, while socio-emotional support from the animal and the amount of animal contact were sometimes associated. Moreover, we found that attachment to and socio-emotional support from the animal were higher in females, in relation to dogs, and when people spent more time with the animal.

**Discussion:**

We found little evidence for a general association between pet ownership or regular animal contact and human health and well-being. However, relationship-related factors, including socio-emotional support from the animal and the amount of contact with the animal, were sometimes associated with well-being. Moreover, the quality of the human–animal relationship depended on different factors, such as the amount of spent time with the animal or animal species. These findings suggest that associations between pet ownership and human health and well-being depend on characteristics of the owner, the animal and the human–animal relationship itself. Our study shows that pet ownership is a complex construct and should be conceptualised multidimensionally to understand its possible positive or negative associations with health outcomes.

## Introduction

Human health and well-being depend on social connection, social support, and socio-demographic factors [1–9], whereas isolation, loneliness, and living alone increase mortality between 25% and 30% [5,10] with pooled effect sizes of 1.32 for social isolation and 1.14 for loneliness [11]. While there is consensus that social connection among members of the same species is central to health and well-being – particularly in social species like humans – less research has looked at cross-species social connections. For many people, animals have a highly emotional meaning [12] and are important social partners [13]. Pets are viewed as integral members of the family [14,15] and can provide significant social support and companionship [16–20]. In Switzerland, 39% of households have pets [21], which is comparable to the mean percentage of 46% in the EU [22], while over 60% of households in the US have pets [23–25]. Thus, for a large portion of Western populations, pets are part of their social network and everyday life.

Although it is commonly assumed that pet ownership is beneficial to human well-being and health – this is sometimes referred to as the “pet effect” [26–28] – the research results are inconsistent. Several studies have confirmed a positive relationship between pet ownership and human well-being [29]. For example, pet owners have reported fewer visits to physicians, better cardiovascular health, and better sleep compared to non-pet owners [30–32]. Meta-analyses have shown that dog owners have a 24% lower mortality rate [33] and a lower risk for cardiovascular diseases [33–35]. Pet ownership has also been linked to greater physical activity, maintained participation in more daily activities, and less cognitive decline in older adults compared to non-pet-owning older adults [36–38]. Moreover, positive associations have also been observed for psychological well-being, higher self-compassion, higher positive affect, as well as lower levels of anxiety, stress reactivity, loneliness, and depression [7,18,39–45]. Owning pets has been found to enhance feelings of autonomy and competence, self-esteem, and life satisfaction [18,45–48]. During the COVID-19 pandemic, animals were frequently perceived as an important source of social support, and pet ownership was associated with less loneliness, depression, anxiety, and decline in mental health during periods of social isolation and the lockdown [49–55]. In particular, dogs seem to have provided owners with a stronger sense of social support compared to potential dog owners who wished to have a dog [51]. Furthermore, owning pets can also expand one’s social network by facilitating social interaction, such as in interacting with people on the street while walking a dog or by providing a conversation topic [30,45].

However, other studies have found no association between pet ownership and human health or well-being or have identified mixed results [7,45,56–66]. For example, some studies have found no association between dog ownership and all-cause or cardiovascular-disease mortality [67], BMI [68], or obesity [69]. There are studies that have found no differences between people with and without pets in their frequency of doctor visits, their number of medications, or their number of hospitalizations [70,71]. Moreover, prospective and longitudinal studies have shown that there is no reduction in loneliness or a change in depression in new pet owners compared to non-pet owners [68,72–74]. In a large representative dataset of American adolescents, prosocial behaviours and the size of peer networks were not associated with pet ownership [75]. And while a recent meta-analysis found that pets have a positive effect on their owner’s mental health compared to non-pet owners, the effect size was low [36]. Also, studies have found that there was no difference in depression, negative emotions, stress, and anxiety or general mental health between pet owners and non-pet owners during the COVID-19 pandemic [55,76].

Some studies have even shown negative associations with pet ownership, such as recurrent falls and fractures in elderly people, dog bites, disease transmission, asthma and other allergies, depression, a higher incidence of heart attacks, and readmissions in heart attack patients [7,45,77]. In Chinese children, pet ownership is often seen as a risk factor for respiratory health, injuries, and *T. gondii* infection [78]. Some studies have found that pet owners have poorer physical health, a higher use of pain relief medication, and a higher risk of depression or anxiety [71,77,79–81]. Pet owners have also been found to participate less frequently in social, recreational, or cultural activities [82]. It is thus unclear if the responsibility for pets is associated with negative psychological well-being and poorer mental health [19,72,83,84]. A large representative study found that cat ownership was associated with increases in loneliness and in social isolation in men, and dog ownership was associated with decreases in life satisfaction in women [85]. This is in line with a study of community-dwelling older adults showing that animal companionship was associated with significantly lower levels of life satisfaction and higher levels of both loneliness and depression [86]. Another longitudinal study revealed that adolescent pet owners were somewhat lonelier than non-pet owners after the COVID-19 pandemic, while there was no difference between pet owners and non-pet owners before the pandemic [87]. Also, pet owners’ psychological well-being declined during the pandemic, while their physical health stayed constant [88].

This shows that the relationship between pet ownership and the physical and mental health of humans is unclear. Different explanations have been proposed to explain the heterogeneity in findings, including weaknesses in research methodology and the complex nature of human–animal relationships, suggesting that associations between pet ownership and health may depend on multiple interacting factors related to the owner, the animal, and the human–animal relationship. One important explanation concerns socio-demographic differences between pet owners and non-pet owners [89–91]. Authors have raised the question of whether pet ownership leads to changes in health, or whether individuals with better or poorer physical or mental health are more likely to acquire pets. In the latter case, observed associations would not reflect causal effects but rather underlying selection processes. Because health is strongly associated with socio-demographic variables, such as age, gender, education, relationship status, and socioeconomic status, differences between pet owners and non-pet owners may partly account for inconsistencies in previous findings [1,2,4,92,93]. Several population-based studies have shown that pet owners differ systematically from non-pet owners in demographic and socioeconomic characteristics: For example, pet owners have been reported to be younger, more likely to be female, partnered, living in rural areas, and to differ in educational level and employment status [7,79]. Importantly, previous studies have shown that controlling for socio-demographic variables can attenuate or eliminate associations between pet ownership and health and well-being, underscoring the need to account for these factors when investigating this relationship [90].

Heterogeneity in results may also reflect differences in the emotional quality and function of human–animal relationships, as conceptualised by psychological theoretical frameworks, such as attachment processes and social support theory. Loneliness or living alone can have a mediating role driving another selection process [94]. It has been hypothesised that individuals experiencing loneliness, anxiety, or depressive symptoms may be more likely to adopt a pet as a compensatory source of emotional support or form of self-medication, seeking stronger emotional bonds with pets to compensate for a lack of human social support [89]. Accordingly, the intensity of the relationship and the strength of the attachment to the animal may mediate the association between pet ownership and well-being [95,96], and several studies have found a relationship between pet attachment style and mental health [66,97–100]. For example, pet owners in families with more conflicts reported stronger pet attachment [14]. Moreover, stronger attachment to the pet was also associated with higher levels of loneliness and depression in pet owners with low levels of human social support [57]. However, findings remain inconsistent across different social and demographic groups. For example, a study based on a representative American sample concluded that the association between pet ownership, attachment, and health may be complex and vary across different groups of people [101], while another study found that stronger attachment to pets was associated with lower symptoms of anxiety and depression, particularly among women with a history of childhood abuse [96].

Variability in findings may further reflect differences between animal species and patterns of interaction. Several studies suggest that dogs may provide stronger social and emotional benefits than other species: For example, dog owners have reported better health, less stress, fewer psychosomatic symptoms, and greater mental health and social functioning compared to people who do not own a dog [102,103]. Similarly, dog owners living alone were less lonely during the lockdown than non-dog owners living alone, while no effect was found for cat owners [104]. Interaction patterns may also play an important role, as attachment to the pet was associated with the duration of interaction and was highest with dogs [91,104]. More broadly, animal species may play an important role in shaping the relationship between pet ownership and mental health and cognitive functioning [38,84]. Although, findings remain inconsistent across different social and demographic groups: For example, one study found that the beneficial effect of pets during the COVID-19 pandemic was highest for women below the age of 40 with a strong attachment to their pet [50], while another study reported that attachment did not vary between different animal species, but that stronger attachment to the pet was associated with lower health before the COVID-19 lockdown [49].

Besides attachment and social support processes, other psychological and neurobiological mechanisms have been proposed to explain why human–animal relationships may influence well-being. Recent research has emphasised that human–pet attachment may be grounded in a neurobiological system shared by mammals, involving co-regulation of the autonomic nervous system, the hypothalamic-pituitary-adrenal axis, oxytocin, or activation of prefrontal-limbic circuits [105–108]. Pets may provide a sense of safety and stability that can facilitate emotional regulation and social buffering through non-conditional and low-threat emotional connection [109–111]. In addition, psychological factors related to the human–pet relationships, such as mindfulness in the presence of pets or feeling authentic during interactions with the pet, seem to influence the association between pet ownership and psychological well-being [112,113].

Taken together, previous research suggests that associations between pet ownership and health may depend on interacting factors related to the owner, the animal, and the quality of the human–animal relationship, including socio-demographic characteristics of the owners, attachment to the animal, perceived socio-emotional support from the animal, the amount of animal contact, and animal species. However, despite repeated calls to identify the moderators and mechanisms underlying the association between pet ownership and human health and well-being, these factors have rarely been examined simultaneously in a single study [91]. To address this research gap, the present study had three aims. First, we investigated differences in health, human social support, life satisfaction, loneliness, and psychological well-being according to pet ownership and regular animal contact status. Second, we examined whether these health- and well-being-related outcomes were associated with the attachment to the animal, the perceived social support from the animal, the animal species, and the amount of animal contact. And third, we investigated whether the animal species and the amount of animal contact are associated with the attachment to the animal and the perceived socio-emotional support from the animal. To address these aims, we analysed a large and nationally representative dataset provided by FORS, the Swiss Centre of Expertise in the Social Sciences, and controlled for different socio-demographic factors that have been identified as important in previous literature.

## Methods

### Study design

The study was based on a regularly conducted cross-sectional survey of the Swiss population called MOSAiCH (Measurement and Observation of Social Attitudes in Switzerland [CH]; [114]). MOSAiCH is connected to the current module of the International Social Survey Programme (ISSP), which has an international and a country-specific part. Participants were drawn from a probabilistic sample representing the Swiss population aged 18 and older.

### Procedures

The results were based on a nationally representative sample of 3,349 permanent residents aged 18 and over who were living in private households in Switzerland. MOSAiCH was conducted by FORS, the Swiss Centre of Expertise in the Social Sciences based in Lausanne, Switzerland (www.forscenter.ch). Data from the survey is publicly available at www.swissubase.ch [115].

The survey consisted of two parts. The sampling of part 1, the ISSP part, took place between 26.02.2021 and 30.06.2021, while the follow-up survey sampling (part 2) started on 07.05.2021 and ended on 31.07.2021. The MOSAiCH sample was randomly drawn from the national population register (SRPH; German: Stichprobenrahmen für Personen- und Haushaltserhebungen) by the Swiss Federal Statistical Office. As of 31.12.2020, Switzerland had a total population of 7,114,731 permanent residents. MOSAiCH was designed to achieve a net sample of 1,000 participants for the follow-up interviews in part 2 of the survey.

Participants were contacted in writing or via email by FORS. All questions were implemented both online (Qualtrics-based) and on paper, and the survey could be conducted in German, French, or Italian. For a detailed description of the recruitment and sampling process, see Ernst Stähli and colleagues [114].

A total of 6,008 individuals were contacted for part 1, resulting in 5,991 eligible cases. A response rate of 55.7% (*N* = 3,349) was achieved. All the respondents who completed at least 50% of the web questionnaire or submitted a valid paper response (not blank) in part 1 were invited to part 2. Of the 3,259 participants who were contacted for the follow-up, 2,328 provided valid interviews (response rate of 38.7%; 69.5% of the respondents from part 1 also participated in part 2).

### Measures

#### MOSAiCH.

MOSAiCH is a cross-sectional survey that focuses on the Swiss population’s values and attitudes towards a wide range of social issues [114]. MOSAiCH was started in 2005 and conducted every two years as a face-to-face survey until 2017. Since 2018, it has been carried out once a year as a self-administered survey. The thematic focus is on the current module of the ISSP (http://www.issp.org). The 2021 survey had two parts. Part 1 contained the 2021 ISSP questionnaire “Health and Health Care” and a socio-demographic part that measures standard variables on household composition, education, occupational situation, religious affiliation, electoral and other forms of participation, and social origin. From this part, we chose the items for health behaviour, healthcare use, human social support use, life satisfaction, loneliness, and psychological well-being. The item with the highest correlation for each construct was selected for this study.

*Health status, health behaviour, and healthcare use.* Health status was operationalised by the self-reported general health status, ranging from 1 (*excellent*) to 5 (*poor*). Health behaviour was measured by the number of smoked cigarettes per day, values ranging from 1 *(do not smoke and never have*) to 7 (*more than 40 cigarettes per day*). Healthcare use was measured by the number of visits to a physician in the last 12 months, with values from 1 (*never*) to 5 (*very often*).

*Human social support.* Social support by a human was measured by the rating of how often someone helps with daily household chores, ranging from 1 (*none of the time*) to 7 (*all of the time*).

*Life satisfaction.* Life satisfaction was measured by how strongly participants perceived their actions as meaningless, ranging from 0 (*does not apply at all*) to 6 (*applies completely*).

*Loneliness.* Loneliness was measured by how often someone felt isolated from other people, ranging from 1 (*never*) to 5 (*very often*).

*Psychological well-being.* Psychological well-being was measured by how often participants felt unable to overcome problems, rated on a scale from 1 (*never*) to 5 (*very often*).

We also extracted the following socio-demographic variables from this part of the questionnaire: gender, age, marital status, education, employment status, household income, partnership status, the number of people in one household, and settlement type. These socio-demographic variables were chosen as previous literature has identified those as important influencing factors for human physical and mental health [1,2,4,7,79,92,93].

Part 2 of MOSAiCH contained questions suggested by researchers from the Swiss research community. They were selected through a competitive call published in January 2020. Six proposals were accepted, one of those containing the data presented here on pet ownership and relationships to pets, the “pet module”.

#### Pet module.

We suggested a set of questions to include in the 2021 MOSAiCH, which were then reviewed by the MOSAiCH scientific commission. Revision was done together with the scientific commission and the authors. The revised questions were translated into German, Italian, and French by two professional translators. All the translations were then reviewed following the TRAPD (Translation, Review, Adjudication, Pretesting, and Documentation) procedure [116] to ensure consistency across languages and alignment with the overall survey.

In the literature, the term *pet* is used inconsistently. Some authors advocate for replacing the term *pet* with *companion animal*, which is another common term used in the literature on human–animal interaction. Hart [117] outlined the diversity of the definitions of the term *pet*. Conservative definitions of *pet* include only dogs and cats. Other authors also include non-domesticated but tamed animals, such as parrots. While some authors include only animals living in the same household as the owner, others also consider animals living outside the household, such as horses or chickens, to be pets. In our study, we defined *pet* as an animal living in the same household with a person. We also thus included non-domesticated animals, such as birds or pet insects. As Hayama, Chang, Gumus, King, and Ernst [118] showed, pet ownership cannot be unequivocally answered with a single question. We therefore assessed pet ownership (defined as living with an animal in the household) and also regular contact with animals outside the household with two questions each. We thus used a dimensional assessment of pet ownership and animal contact and further assessed the type of animal, average time spent with an animal per day, amount of contact with the closest animal, as well as socio-emotional support from and attachment to the animal.

The following constructs and items were used for the pet module:

*Pet ownership and regular animal contact*. Six items covered pet ownership and if a person had regular animal contact:

(a) Whether participants were living with one or more animals in their household (currently/ in the last 6 months but not now/ no).
(b) Animal species in the household (based on response to item a).
(c) Whether participants had regular contact with one or more animals outside the household in the past 6 months (yes at work/ yes in my leisure time/ yes in other situations [specify]/ no).
(d) Animal species participants had contact with outside the household (based on response to item c).
(e) Average time spent with animals per day (more than 2 hours/ 1–2 hours/ 30–60 min/ 10–30 min/ less than 10 min).
(f) Whether the animal felt closest to was living in the household or not (household/ not in my household).

The species options for items b and d included: cat, dog, small mammals (e.g., rabbit, hamster, guinea pig), bird, fish, reptile (e.g., snake, lizard, tortoise), insect, amphibian, or gastropod (e.g., snail, frog, newt, toad, salamander), equine (horse, pony, donkey), farm animal (e.g., cow, sheep, goat, chicken), and other animal species (to be specified by participants).

The following items were reported by participants in relation to the animal they felt closest to.

*Amount of animal contact.* People indicated the average amount of time they spent with their closest animal, on a scale from 6 (*more than 2 hours per day*) to 1 (*0 minutes per day*).

*Socio-emotional support from animal.* To select relevant items capturing perceived socio-emotional support from the closest animal, we reviewed existing questionnaires used in studies on social and emotional support provided by pets and listed potential items. Then we compared these items with items measuring human social support from the 2021 MOSAiCH survey (NQ31–NQ36) to ensure the highest possible comparability. We selected items from the validated Comfort from Companion Animal Scale by Zasloff [119], the validated Lexington Attachment to Pets Scale by Johnson, Garrity, and Stallones [120], the validated Owner-Pet Relationship Scale by Winefield, Black, and Chur-Hansen [64], and the items included in the study by Kurdek [121]. Further selection was made based on wording. Attention was paid to the fact that all items could be answered following the same response pattern and that the statements were about the participant and not about the animal (e.g., “my pet loves me” vs. “my pet makes me feel loved”). Next, the items were adjusted from “my pet” to “this animal” to ensure that the statements also applied to non-pet owners who have regular contact with animals.

The following items were included in the questionnaire, both rated on a scale from 0 (*does not apply at all*) to 6 (*applies completely*), with higher scores indicating higher agreement:

a) “This animal helps me get through tough times.” (Measure for emotional support: safe haven; adapted from Kurdek [121] and the Owner-Pet Relationship Scale [item 5] by Winefield et al., [64]).
b) “This animal makes me feel loved and trusted.” (Measure for emotional support: secure base; adapted from the Comfort from Companion Animal Scale [items 12 and 13] by Zasloff [119]).

*Attachment to animal.* To select relevant items for measuring perceived attachment to the closest animal, we conducted a literature review of existing questionnaires about pet attachment and listed potential items. We then chose items that cover the following dimensions of attachment defined by Ainsworth [122]: safe haven, secure base, and separation distress (items Q7a, Q7b, Q7c). Because the dimension of safe haven overlaps with emotional support, an additional item to measure attachment was not included, as this is already included in the concept of socio-emotional support from the animal. Further, we included a self-report item on emotional closeness with the animal, as this item has exhibited good differentiation in prior research [120]. The included items were from the Comfort from Companion Animal Scale, the Lexington Attachment to Pets Scale, the Owner-Pet Relationship Scale, and from the items included in the study by Kurdek [121]. The following items measuring attachment to the closest animal were included, each rated on a scale from 0 (*does not apply at all*) to 6 (*applies completely*), with higher scores indicating agreement:

a) “I think about this animal when it is not with me.” (Measure for separation distress; adapted from the Owner-Pet Relationship Scale [item 13] by Winefield et al., [64]).
b) “I feel this animal is part of my family.” (Measure for secure base; adapted from Lexington Attachment to Pets Scale [item t] by Johnson et al. [120] and from Owner-Pet Relationship Scale [item 7] by Winefield et al., [64]).
c) “This animal and I have a very close relationship.” (Measure of closeness; source: Lexington Attachment to Pets Scale [item o] by Johnson et al., [120]).

### Data analyses

All analyses were based on the dataset retrieved from FORS [115]. The data of the online questionnaires was coded directly by the Qualtrics survey software, while the data of the paper questionnaires was manually entered and coded in office by FORS. Data checking and cleaning was also conducted by FORS; the detailed procedure can be found in the publication by Ernst Stähli and colleagues [114].

Theoretical models with outcome variables and predictors were prespecified according to hypotheses and findings from previous literature. For each variable, one questionnaire item was chosen to be included in the model. Since each construct, such as health behaviour, was measured by different items in the MOSAiCH questionnaire, we built a sum score out of all the items for each variable construct and chose the item for each construct that had the highest correlation with that score. Then we used lasso-regularised regression using the glmnet package [123] for each prespecified theoretical model to select the variables for the final models using an available case approach (*n* = 1,137). To select the optimal regularization parameter (λ), we employed 10-fold cross-validation via the cv.glmnet function. The best λ value was determined using lambda.min, which corresponds to the λ that minimizes the cross-validation error. Each model was run 10 times, and if an item was included in five or more of the runs, we included it in the final model. For these final models, we imputed missing data with the mice package [124]. In a next step, we used linear models to investigate the effect of the remaining factors within each model.

The following models were tested with data from all participants (*N* = 2,328) to investigate the differences in health status, health behaviour, healthcare use, psychological well-being, life satisfaction, loneliness, and human social support by pet ownership and regular animal contact status (question 1). The original models for health had health status (model 1.1), health behaviour (model 1.2), and healthcare use (model 1.3) as the dependent variables, while pet ownership, regular animal contact, loneliness, psychological well-being, and human social support were the predictors. These predictors were chosen based on literature showing that human health highly depends on perceived loneliness, human social support and psychological well-being [92,125]. All the models included gender, age, marital status, education, employment status, household income, partnership status, the number of people in one household, and settlement type as the control variables before the lasso procedure. The socio-demographic variables were chosen based on previous literature identifying them as important factors for human health [1,2,4,7,79,92,93]. The original model with loneliness as the dependent variable (model 2) had pet ownership, regular animal contact, and human social support as the predictors, while gender, age, marital status, education, employment status, household income, partnership status, the number of people in one household, and settlement type served as the control variables. The predictor was chosen based on literature showing that loneliness is often closely linked to human social support [126].

The original model for life satisfaction (model 3) had pet ownership, regular animal contact, loneliness, health status, health behaviour, healthcare use, and human social support as the predictors. Predictors were chosen because they can all be closely linked to each other, as recent studies show [127,128]. Gender, age, marital status, education, employment status, household income, partnership status, the number of people in one household, and settlement type served as the control variables. The original model for psychological well-being (model 4) included pet ownership, regular animal contact, loneliness, and human social support as the predictors, while gender, age, marital status, education, employment status, household income, partnership status, the number of people in one household, and settlement type served as the control variables. The choice of the predictors was based on literature [92,129,130]. The original model for human social support (model 5) included pet ownership and regular animal contact as the predictors, while gender, age, marital status, education, employment status, household income, partnership status, the number of people in one household, and settlement type served as the control variables. All the final models with the respective items and the reduction process can be found in S1 Supporting information (Tables S1–S7 in S1 File).

To investigate if the attachment to animals, the socio-emotional support from animals, the amount of animal contact and animal species were related to health, loneliness, life satisfaction and psychological well-being (question 2), we ran models with all the participants who indicated that they owned pets or had regular animal contact (*n* = 1,105). The choice of predictors had the same basis as mentioned above. The original models had health status (model 6.1), health behaviour (model 6.2), and healthcare use (model 6.3) as the dependent variables, while loneliness, psychological well-being, human social support, pet ownership, regular animal contact, the number of animal species, the animal species, the amount of animal contact, attachment to the animal, and socio-emotional support from the animal were used as the predictors. The models included gender, age, marital status, education, employment status, household income, partnership status, the number of people in one household, and settlement type as the control variables. The original model for loneliness (model 7) included human social support, pet ownership, regular animal contact, the number of animal species, the animal species, the amount of animal contact, attachment to the animal, and socio-emotional support from the animal as the predictors and gender, age, marital status, education, employment status, household income, partnership status, the number of people in one household, and settlement type as the control variables. The original model for life satisfaction (model 8) included loneliness, health status, health behaviour, healthcare use, human social support, pet ownership, regular animal contact, the number of animal species, the animal species, the amount of contact with animal, attachment to the animal and socio-emotional support from the animal as the predictors with gender, age, marital status, education, employment status, partnership status, the number of people in one household, household income and settlement type as the control variables. The original model for psychological well-being (model 9) included loneliness, human social support, pet ownership, regular animal contact, the number of animal species, the animal species, the amount of animal contact, attachment to the animal, and socio-emotional support from the animal as the predictors, while gender, age, marital status, education, employment status, partnership status, the number of people in one household, household income, and settlement type were the control variables.

To investigate if the attachment to the animal and the socio-emotional support from the animal were related to the animal species and the amount of animal contact (question 3), we ran two models with attachment to the animal (model 10.1) and socio-emotional support from the animal (model 10.2) as the dependent variables. Both models included the amount of animal contact, the number of animal species, the animal species, human social support, and whether the chosen animal lived in the household or outside as the predictors, while gender, age, marital status, education, employment status, partnership status, the number of people in one household, household income, and settlement type were the control variables. All the models with the respective items and the reduction process can be found in S1 Supporting information (Tables S8–S15 in S1 File).

For the predictor marital status, we combined the variables separated, divorced, and widowed into the single category “separated”. For the predictor settlement type, we combined countryside and village to the single category “rural” and combined city and town into the single category “urban” to ensure adequate sample sizes within each category.

In the pet module, we combined the items “currently living with pets” and “lived with pets in the last 6 months but not now” to calculate the total population with pets at the moment or within the last 6 months. We also combined small mammals and birds into one category, animals living in a terrarium or aquarium into one category, and equines and farm animals into one category to ensure adequate sample sizes within each category. We calculated a sum score for socio-emotional support from the animal (two items aggregated, higher values indicating more socio-emotional support) and a sum score for attachment to the animal (three items aggregated, higher values indicating stronger attachment).

We report our outcomes according to the Consolidated Standards of Reporting Trials (CONSORT), which suggest using the estimated coefficient with the confidence interval. The mean difference (coefficient) was used as the effect size, the confidence interval was defined at 95%, and the significance level was set at  .05. All analyses were conducted with R Statistical Software (version, 4.2.1; R Core Team, 2021) using the glmnet package to run all the models [123].

## Results

### Descriptive statistics

From the total sample of 3,349 participants, 2,328 indicated whether they had pets or regular animal contact or not. Out of these, 1,105 participants indicated that they had pets or regular animal contact, while 1,223 participants did not have pets or regular animal contact. Table 1 shows the descriptive statistics of participants with and without animal contact. Table 2 shows the attachment to and the socio-emotional support from the closest animal, as well as the time spent with the different animal species, either for pets living in the household or for animals with whom the participants had regular contact outside the household.

**Table 1:** Descriptive statistics for participants with pets or animal contact and those with neither.

		No pet ownership or no regular animal contact	Pet ownership or regular animal contact (at the moment or within the last 6 months)
*n*		1,223	1,105
Age, *M* (*SD*)		54.09 (17.52)	49.91 (16.56)
Gender, *n* (%)	Men	609 (50%)	473 (43%)
	Women	614 (50%)	632 (57%)
Marital status, *n* (%)	Married	664 (54%)	589 (54%)
	Separated	18 (2%)	18 (2%)
	Divorced	134 (11%)	102 (9%)
	Widowed	64 (5%)	55 (5%)
	Never married	339 (28%)	336 (31%)
	NA	4 (0.3%)	5 (0.5%)
Education, *n* (%)	Basic education	8 (1%)	3 (0.3%)
	Secondary school	44 (5%)	30 (3%)
	Secondary technical school	39 (4%)	29 (3%)
	Gymnasium (Matura)	86 (9%)	87 (10%)
	Vocational education	411 (43%)	448 (51%)
	University of applied sciences or school of education	135 (14%)	92 (10%)
	University or ETH	236 (25%)	182 (21%)
	Other	2 (0.2%)	7 (1%)
	NA	262 (21%)	227 (21%)
Education in years, *M* (*SD*)		12.23 (3.92)	11.88 (3.67)
Employment status, *n* (%)	Employed	676 (57%)	691 (64%)
	Unemployed	27 (2%)	35 (3%)
	In education	30 (3%)	46 (4%)
	Apprentice/trainee	13 (1%)	19 (2%)
	Permanently sick/disabled	10 (1%)	13 (1%)
	Retired	345 (29%)	213 (20%)
	Housework/care	42 (4%)	37 (3%)
	Military or community service	0 (0%)	0 (0%)
	Other	41 (3%)	31 (3%)
	NA	39 (3%)	20 (2%)
Partnership status, *n* (%)	No partner	302 (25%)	252 (23%)
	Partner in same household	812 (67%)	757 (69%)
	Partner in different household	91 (8%)	92 (8%)
	NA	18 (2%)	4 (0.4%)
Number of people in one household, *n* (%)	1 person	226 (19%)	160 (15%)
	2 people	558 (48%)	430 (40%)
	3–5 people	364 (31%)	455 (42%)
	6 or more people	12 (1%)	27 (3%)
	NA	63 (5%)	33 (3%)
Household monthly income in CHF, *n* (%)	Less than 3,300 CHF	73 (8%)	67 (7%)
	3,300–5,299 CHF	188 (19%)	176 (20%)
	5,300–8,799 CHF	330 (34%)	285 (32%)
	8,799–15,599 CHF	314 (32%)	305 (34%)
	15,600 CHF or more	65 (7%)	63 (7%)
	NA	253 (21%)	209 (19%)
Settlement type, *n* (%)	Urban (city/town)	534 (44%)	332 (30%)
	Suburban	167 (14%)	107 (10%)
	Rural (village/countryside)	509 (42%)	655 (60%)
	NA	13 (1%)	11 (1%)
Health status, *M* (*SD*)		2.68 (0.85)	2.68 (0.90)
Health behaviour, *M* (*SD*)		1.82 (1.17)	1.95 (1.28)
Healthcare use, *M* (*SD*)		2.33 (0.98)	2.30 (1.01)
Human social support, *M* (*SD*)		5.48 (1.93)	5.57 (1.83)
Life satisfaction, *M* (*SD*)		2.46 (1.62)	2.50 (1.64)
Loneliness, *M* (*SD*)		2.35 (1.10)	2.32 (1.12)
Psychological well-being, *M* (*SD*)		1.78 (0.99)	1.85 (1.01)

Note: ETH = Swiss Federal Institute of Technology.

**Table 2:** Attachment to the animal, socio-emotional support from the animal, and amount of animal contact by animal species for pet ownership and regular animal contact.

Species		*n*	Attachment, *M* (*SD*)	Socio-emotional support, *M* (*SD*)	Amount of contact, *M* (*SD*)
Living with animal in household	Cat	554	13.28 (4.64)	7.89 (3.75)	4.67 (1.34)
	Dog	303	15.04 (4.00)	9.22 (3.29)	5.44 (0.97)
	Animal kept in enclosure that can be touched (small mammal, bird)	114	10.78 (5.77)	6.29 (4.10)	4.03 (1.54)
	Animal kept in terrarium or aquarium (fish, reptile, insect, amphibian, gastropod)	100	12.43 (5.57)	7.07 (4.22)	4.51 (1.46)
	Farm animal (cow, sheep, goat, chicken) or equine (horse, pony, donkey)	26	14.08 (4.63)	8.48 (3.37)	5.40 (0.91)
Regular contact with animal outside household	Cat	320	11.52 (6.09)	7.10 (4.16)	4.11 (1.61)
	Dog	446	12.44 (5.81)	7.73 (4.03)	4.33 (1.60)
	Animal kept in enclosure that can be touched (small mammal, bird)	85	11.10 (5.98)	7.21 (4.06)	4.43 (1.48)
	Animal kept in a terrarium or aquarium (fish, reptile, insect, amphibian, gastropod)	66	11.39 (5.63)	6.94 (3.78)	4.61 (1.52)
	Farm animal (cow, sheep, goat, chicken) or equine (horse, pony, donkey)	96	11.93 (5.79)	7.45 (3.92)	4.90 (1.41)
	Other	98	11.86 (5.94)	7.38 (3.96)	4.76 (1.47)

Note: Attachment to the animal ranges from 0 to 18; social support from the animal ranges from 0 to 12; amount of animal contact ranges from 1 to 6.

The descriptive statistics show that pet owners or people with regular animal contact were, in general, four years younger and were more likely to be female compared to non-pet owners and people without regular animal contact. Their marital status was comparable, while pet owners or people with regular animal contact had completed vocational training slightly more often but possessed a degree from a university or a university of applied sciences less often. Pet owners or people with regular animal contact were also more likely to be employed or to be in education, and were less likely to be retired compared to non-pet owners and people without regular animal contact. The presence of a partner and household income were comparable, while pet owners or people with regular animal contact were less likely to live in a household with one or two people and more likely to live in a household with three or more people. Moreover, pet owners or people with regular animal contact were less likely to live in a city, suburb, or town and more likely to live in a village or the countryside than non-pet owners and people without regular animal contact.

### Differences in health status, health behaviour, healthcare use, psychological well-being, life satisfaction, loneliness, and human social support by pet ownership and regular animal contact status

#### Health status.

People with pets had a significantly lower self-reported health status compared to people without pets when we controlled for gender, age, marital status, employment status, household income, partnership status, the number of people in the household, and settlement type (see Table 3). The effect was, however, small. People with regular animal contact and people without regular animal contact did not differ in their health status.

**Table 3:** Health status, health behaviour, and healthcare use by pet ownership and regular animal contact status.

Outcome	Parameter	Coefficient	95% CI	*p* value
Health status	Animal in household	0.08	0.00 to 0.15	.038*
	Regular animal contact	−0.02	−0.10 to 0.06	.658
	Loneliness	0.03	0.00 to 0.06	.029*
	Well-being	0.29	0.25 to 0.32	<.001***
	Human social support	−0.01	−0.03 to 0.01	.234
	Gender	−0.10	−0.17 to −0.03	.004**
	Age	0.01	0.01 to 0.01	<.001***
	Never married	0.07	−0.03 to 0.17	.180
	Employed	0.06	−0.07 to 0.19	.367
	Unemployed and looking for job	0.10	−0.14 to 0.34	.412
	Permanently sick or disabled	0.97	0.62 to 1.33	<.001***
	Retired	0.16	−0.01 to 0.33	0.068
	Doing housework, looking after the home, children, or other people	0.15	−0.08 to 0.38	.191
	Household income	−0.03	−0.05 to −0.01	<.001***
	No partner	0.06	−0.04 to 0.15	.245
	Partner in different household	−0.09	−0.22 to 0.04	.172
	Number of people in household	−0.03	−0.06 to 0.00	.064
	Suburban	0.08	−0.03 to 0.18	.155
	Urban (city, town)	0.01	−0.06 to 0.09	.682
Health behaviour	Animal in household	0.19	0.08 to 0.30	.001***
	Regular animal contact	0.01	−0.11 to 0.13	.858
	Loneliness	0.02	−0.03 to 0.07	.396
	Well-being	0.08	0.03 to 0.14	.002**
	Human social support	0.02	−0.01 to 0.04	.130
	Gender	−0.19	−0.29 to −0.09	<.001***
	Married	−0.06	−0.02 to 0.07	.376
	Separated (incl. divorced, widowed)	0.05	−0.11 to 0.21	.548
	Education in years	−0.04	−0.05 to −0.02	<.001***
	Employed	−0.06	−0.38 to 0.26	.724
	Unemployed and looking for job	0.08	−0.35 to 0.51	.715
	In education (unpaid)	−0.39	−0.81 to 0.02	.064
	Apprentice or trainee	−0.61	−1.15 to −0.07	.028*
	Permanently sick or disabled	0.42	−0.25 to 1.09	.219
	Retired	−0.24	−0.57 to 0.09	.146
	Doing housework, looking after the home, children, or other people	−0.35	−0.77 to 0.07	.100
	Household income	−0.03	−0.06 to −0.01	.003**
	Partner in different household	0.07	−0.12 to 0.26	.470
	Number of people in household	−0.03	−0.07 to 0.02	.217
	Rural (countryside, village)	−0.13	−0.25 to −0.02	.019*
	Suburban	−0.01	−0.18 to 0.16	.910
Healthcare use	Animal in household	0.03	−0.06 to 0.12	.506
	Regular animal contact	0.02	−0.08 to 0.11	.739
	Well-being	0.20	0.16 to 0.24	<.001***
	Age	0.01	0.00 to 0.01	<.001***
	Separated (incl. divorced, widowed)	0.09	−0.03 to 0.20	.159
	Education in years	0.00	−0.01 to 0.01	.766
	Employed	−0.09	−0.32 to 0.14	.434
	Unemployed and looking for job	0.07	−0.25 to 0.39	.654
	In education (unpaid)	−0.11	−0.43 to 0.21	.512
	Apprentice or trainee	0.40	−0.01 to 0.80	.055
	Permanently sick or disabled	0.72	0.29 to 1.15	.001***
	Retired	0.15	−0.09 to 0.40	.218
	Doing housework, looking after the home, children, or other people	−0.17	−0.47 to 0.14	.287
	No partner	−0.02	−0.13 to 0.08	.694
	Rural (countryside, village)	−0.06	−0.18 to 0.07	.383
	Urban (city, town)	0.05	−0.08 to 0.18	.448

Note: CI: 95% confidential interval, * indicates *p* ≤ .05, ** indicates *p* ≤ .01, *** indicates *p* ≤ .001.

#### Health behaviour.

Pet owners reported a significantly but only slightly higher frequency of unhealthy behaviour (measured as smoking 0.19 cigarettes more per day) compared to people who did not own pets when we controlled for gender, education, marital status, employment status, household income, partnership status, the number of people in the household, and settlement type (see Table 3). People with regular animal contact and people without regular animal contact did not differ in their health behaviour.

#### Healthcare use.

People with pets did not differ in their self-reported healthcare use measured in the number of visits to a physician compared to people without pets when we controlled for age, education, marital status, employment status, household income, partnership status, the number of people in the household, and settlement type (see Table 3). Moreover, people with and without regular animal contact did not differ in their healthcare use.

#### Loneliness.

Pet owners did not differ in their self-reported loneliness compared to non-pet owners when we controlled for age, education, employment status, the number of people living in the household, and settlement type (see Table 4). Moreover, people with and without regular animal contact did not differ in self-reported loneliness.

**Table 4:** Loneliness, life satisfaction, psychological well-being, and human social support by pet ownership and regular animal contact status.

Outcome	Parameter	Coefficient	95% CI	*p* value
Loneliness	Animal in household	−0.01	−0.11 to 0.09	.831
	Regular animal contact	0.00	−0.11 to 0.10	.962
	Human social support	−0.05	−0.07 to −0.03	<.001***
	Age	−0.01	−0.01 to 0.00	<.001***
	Education in years	0.00	−0.01 to 0.02	.582
	Employed	−0.10	−0.22 to 0.01	.087
	Unemployed and looking for job	0.21	−0.08 to 0.50	.156
	In education (unpaid)	0.20	−0.10 to 0.50	.198
	Number of people in household	−0.03	−0.07 to 0.00	.082
	Rural (countryside, village)	−0.04	−0.14 to 0.06	.468
	Suburban	0.15	0.00 to 0.30	.044*
Life satisfaction	Animal in household	0.06	−0.08 to 0.20	.402
	Regular animal contact	0.01	−0.14 to 0.16	.899
	Loneliness	0.32	0.27 to 0.38	<.001***
	Health status	0.32	0.24 to 0.39	<.001***
	Age	−0.01	−0.01 to 0.00	.006**
	Never married	0.17	−0.01 to 0.35	.070
	Education in years	−0.02	−0.04 to −0.01	.012*
	Employed	−0.22	−0.37 to −0.06	.005**
	Unemployed and looking for job	0.22	−0.19 to 0.64	.286
	Permanently sick or disabled	−0.06	−0.69 to 0.57	.848
	Partner in same household	−0.02	−0.18 to 0.14	.802
	Rural (countryside, village)	−0.15	−0.29 to −0.01	.042*
	Suburban	0.18	−0.04 to 0.39	.109
Psychological well-being	Animal in household	0.05	−0.04 to 0.13	.284
	Regular animal contact	0.00	−0.09 to 0.09	.926
	Loneliness	0.19	0.15 to 0.22	<.001***
	Human social support	−0.03	−0.05 to −0.01	.002**
	Gender	0.08	0.00 to 0.16	.044*
	Age	−0.01	−0.01 to −0.01	<.001***
	Never married	0.02	−0.09 to 0.14	.720
	Education in years	−0.01	−0.02 to 0.00	.054
	Employed	−0.21	−0.31 to −0.11	<.001***
	Unemployed and looking for job	0.41	0.15 to 0.66	.002**
	Permanently sick or disabled	0.43	0.04 to 0.81	.030*
	Doing housework, looking after the home, children, or other people	−0.42	−0.64 to −0.19	<.001***
	Household income	−0.02	−0.04 to 0.00	.046*
	No partner	0.10	−0.06 to 0.26	.207
	Partner in same household	−0.01	−0.17 to 0.14	.851
	Number of people in household	0.03	−0.01 to 0.06	.108
	Suburban	0.03	−0.09 to 0.16	.603
	Urban (city, town)	0.15	0.07 to 0.24	.001***
Human social support	Animal in household	0.04	−0.05 to 0.13	.396
	Regular animal contact	0.01	−0.09 to 0.10	.886
	Gender	0.10	0.02 to 0.18	.019*
	Age	−0.01	−0.01 to −0.01	<.001***
	Separated (incl. divorced, widowed)	0.05	−0.08 to 0.17	.484
	Employed	−0.23	−0.46 to −0.01	.043*
	Unemployed and looking for job	0.48	0.15 to 0.81	.004**
	In education (unpaid)	0.16	−0.16 to 0.47	.329
	Apprentice or trainee	0.20	−0.21 to 0.61	.336
	Permanently sick or disabled	0.44	0.00 to 0.88	.049*
	Retired	−0.09	−0.34 to 0.16	.477
	Doing housework, looking after the home, children, or other people	−0.41	−0.71 to −0.10	.009**
	Household income	−0.02	−0.04 to −0.01	.006**
	No partner	0.14	−0.02 to 0.30	.079
	Partner in same household	0.01	−0.15 to 0.17	.882
	Rural (countryside, village)	−0.15	−0.24 to −0.06	.001***
	Suburban	−0.08	−0.21 to 0.05	.235

Note: CI: 95% confidential interval, * indicates *p* ≤ .05, ** indicates *p* ≤ .01, *** indicates *p* ≤ .001.

#### Life satisfaction.

People with pets did not differ in their self-reported life satisfaction compared to people without pets when we controlled for age, marital status, education, employment status, partnership status, and settlement type (see Table 4). Moreover, people with and without regular animal contact did not differ in self-reported life satisfaction.

#### Psychological well-being.

Pet owners did not differ in their self-reported psychological well-being compared to non-pet owners when we controlled for gender, age, marital status, education, employment status, household income, partnership status, the number of people in the household, and settlement type (see Table 4). Moreover, people with and without regular animal contact did not differ in self-reported psychological well-being.

#### Human social support.

People with pets did not differ in their self-reported human social support compared to people without pets when we controlled for gender, age, marital status, employment status, household income, partnership status, and settlement type (see Table 4). Moreover, people with and without regular animal contact did not differ in human social support.

Taken together, these findings show that pet owners reported a significantly lower self-reported health status and a slightly higher frequency of unhealthy behaviour compared to people without pets. People with and without regular animal contact did not differ in any of the health or well-being outcomes.

### Attachment to animals, socio-emotional support from animals, amount of animal contact and animal species related to health, loneliness, life satisfaction and psychological well-being

#### Health status.

Attachment to the animal and the amount of animal contact did not have a relationship with health status when we controlled for gender, age, marital status, employment status, household income, partnership status, the number of people in the household, and settlement type (see Table 5). No relationship was found for having small mammals or birds or farm animals or equines as pets, nor was one found for having regular contact with cats or with animals kept in a terrarium or aquarium. The number of animal species and socio-emotional support from the closest animal were not included in the model after the lasso selection.

**Table 5:** Attachment to animals, socio-emotional support from animals, amount of animal contact and animal species related to health.

Outcome	Parameter	Coefficient	95% CI	*p* value
Health status	Loneliness	0.05	0.01 to 0.10	.026*
	Well-being	0.28	0.23 to 0.33	<.001***
	Human social support	0.00	−0.03 to 0.02	.970
	Animal in household	0.05	−0.10 to 0.21	.518
	Regular animal contact	−0.04	−0.17 to 0.09	.534
	Amount of animal contact	0.01	−0.03 to 0.05	.742
	Animal kept in enclosure that can be touched (small mammal, bird) in household	−0.06	−0.23 to 0.11	.478
	Farm animal (cow, sheep, goat, chicken) or equine (horse, pony, donkey) in household	0.24	−0.08 to 0.57	.139
	Regular contact with cat	−0.05	−0.17 to 0.08	.460
	Regular contact with animal kept in terrarium or aquarium (fish, reptile, insect, amphibian, gastropod)	0.12	−0.10 to 0.33	.287
	Attachment to animal	−0.01	−0.02 to 0.00	.199
	Gender	−0.10	−0.02 to −0.00	.046*
	Age	0.01	0.01 to 0.02	<.001***
	Never married	0.10	−0.04 to 0.24	.167
	Unemployed and looking for job	−0.03	−0.32 to 0.26	.834
	In education (unpaid)	−0.07	−0.33 to 0.19	.601
	Permanently sick or disabled	1.23	0.74 to 1.71	<.001***
	Household income	−0.03	−0.05 to 0.00	.027*
	No partner	0.07	−0.06 to 0.21	.294
	Number of people in household	−0.03	−0.07 to 0.00	.091
	Suburban	0.21	0.04 to 0.37	.013*
	Urban (city, town)	−0.05	−0.16 to 0.06	.387
Health behaviour	Human social support	0.03	0.00 to 0.07	.088
	Animal in household	0.08	−0.15 to 0.31	.488
	Regular animal contact	0.05	−0.31 to 0.24	.577
	Amount of animal contact	0.08	0.02 to 0.13	.008**
	Farm animal (cow, sheep, goat, chicken) or equine (horse, pony, donkey) in household	−0.39	−0.89 to 0.12	.135
	Regular contact with farm animal (cow, sheep, goat, chicken) or equine (horse, pony, donkey)	−0.23	−0.51 to 0.06	.117
	Gender	−0.18	−0.34 to −0.03	.021*
	Education in years	−0.04	−0.07 to −0.02	<.001***
	Employed	0.24	0.06 to 0.14	.008**
	Permanently sick or disabled	0.58	−0.15 to 1.31	.118
	Doing housework, looking after the home, children, or other people	−0.27	−0.71 to 0.17	.226
	Household income	−0.04	−0.07 to 0.00	.028*
	Number of people in household	−0.03	−0.08 to 0.02	.227
Healthcare use	Well-being	0.20	0.14 to 0.26	<.001***
	Animal in household	−0.02	−0.20 to 0.16	.812
	Regular animal contact	−0.03	−0.17 to 0.11	.673
	Amount of animal contact	−0.02	−0.07 to 0.03	.395
	Regular contact with farm animal (cow, sheep, goat, chicken) or equine (horse, pony, donkey)	−0.06	−0.27 to 0.16	.618
	Socio-emotional support from animal	0.01	0.00 to 0.03	.157
	Age	0.01	0.00 to 0.01	.019*
	Separated (incl. divorced, widowed)	0.09	−0.10 to 0.27	.356
	Education in years	0.00	−0.02 to 0.01	.796
	Employed	0.04	−0.19 to 0.28	.717
	Unemployed and looking for job	0.20	−0.19 to 0.60	.314
	In education (unpaid)	0.08	−0.30 to 0.47	.669
	Apprentice or trainee	0.63	0.11 to 1.14	.017*
	Permanently sick or disabled	1.17	0.60 to 1.75	<.001***
	Retired	0.25	−0.03 to 0.54	.081
	No partner	0.01	−0.15 to 0.17	.899
	Rural (countryside, village)	−0.15	−0.35 to 0.05	.151
	Urban (city, town)	−0.02	−0.23 to 0.20	.888

Note: CI: 95% confidential interval, * indicates *p* ≤ .05, ** indicates *p* ≤ .01, *** indicates *p* ≤ .001.

#### Health behaviour.

A higher amount of animal contact was significantly associated with health behaviour, as a slightly higher number of cigarettes were smoked per day when we controlled for gender, education, employment status, household income, and the number of people in the household (see Table 5). Having farm animals or equines as pets or having regular contact with farm animals or equines did not have a significant relationship with health behaviour. The number of animal species, attachment to the animal, and socio-emotional support from the animal were not included in the model after the lasso selection.

#### Healthcare use.

The amount of animal contact and the socio-emotional support from the animal were not associated with healthcare use when we controlled for age, marital status, education, employment status, partnership status, and settlement type (see Table 5). We also did not find any association for regular contact with farm animals or equines. Other species, the number of animal species, and attachment to the animal were not included after the lasso selection.

#### Loneliness.

The amount of animal contact and having animals in a terrarium or aquarium were not associated with loneliness when we controlled for age, education, employment status, the number of people in the household, and settlement type (see Table 6). Higher socio-emotional support from the animal was tendentially associated with slightly higher loneliness. The number of animal species, other species, and attachment to the animal were not included after the lasso selection.

**Table 6:** Attachment to animals, socio-emotional support from animals, amount of animal contact, and animal species related to loneliness, life satisfaction, and psychological well-being.

Outcome	Parameter	Coefficient	95% CI	*p* value
Loneliness	Human social support	−0.05	−0.09 to −0.02	.001***
	Animal in household	0.00	−0.20 to 0.21	.982
	Regular animal contact	0.02	−0.13 to 0.18	.790
	Amount of animal contact	0.00	−0.06 to 0.05	.904
	Animal kept in terrarium or aquarium (fish, reptile, insect, amphibian, gastropod) in household	−0.13	−0.36 to 0.10	.277
	Socio-emotional support from animal	0.02	0.00 to 0.04	.083
	Age	−0.01	−0.01 to 0.00	.006**
	Education in years	0.01	−0.01 to 0.03	.161
	Employed	−0.11	−0.28 to 0.05	.184
	Unemployed and looking for job	0.16	−0.23 to 0.56	.419
	In education (unpaid)	0.31	−0.08 to 0.70	.124
	Number of people in household	−0.03	−0.07 to 0.02	.243
	Rural (countryside, village)	−0.10	−0.25 to 0.05	.196
	Suburban	0.09	−0.15 to 0.33	.473
Life satisfaction	Loneliness	0.33	0.24 to 0.41	<.001***
	Human social support	0.33	0.22 to 0.44	<.001***
	Animal in household	0.13	−0.15 to 0.41	.363
	Regular animal contact	0.06	−0.16 to 0.29	.589
	Amount of animal contact	−0.04	−0.11 to 0.03	.265
	Regular contact with other species	−0.47	−0.81 to −0.13	.007**
	Age	−0.01	−0.01 to 0.00	.050*
	Never married	0.18	−0.08 to 0.45	.175
	Education in years	−0.02	−0.04 to 0.01	.235
	Employed	−0.12	−0.34 to 0.10	.294
	Unemployed and looking for job	0.43	−0.12 to 0.99	.128
	Permanently sick or disabled	0.01	−0.90 to 0.92	.988
	Partner in same household	−0.09	−0.31 to 0.14	.452
	Rural (countryside, village)	−0.22	−0.43 to −0.01	.045*
	Suburban	0.11	−0.23 to 0.45	.532
Psychological well-being	Loneliness	0.21	0.16 to 0.26	<.001***
	Human social support	−0.05	−0.08 to −0.03	<.001***
	Animal in household	−0.01	−0.19 to 0.16	.887
	Regular animal contact	−0.13	−0.32 to 0.05	.162
	Amount of animal contact	−0.04	−0.09 to 0.00	.076
	Dog in household	0.08	−0.07 to 0.23	.284
	Animal kept in terrarium or aquarium (fish, reptile, insect, amphibian, gastropod) in household	0.13	−0.07 to 0.33	.204
	Farm animal (cow, sheep, goat, chicken) or equine (horse, pony, donkey) in household	−0.18	−0.55 to 0.20	.352
	Regular contact with dog	0.03	−0.14 to 0.19	.735
	Regular contact with animal kept in enclosure that can be touched (small mammal, bird)	0.16	−0.06 to 0.38	.156
	Regular contact with animal kept in terrarium or aquarium (fish, reptile, insect, amphibian, gastropod)	0.33	0.07 to 0.58	.011*
	Regular contact with other species	−0.05	−0.27 to 0.16	.620
	Socio-emotional support from animal	0.01	0.00 to 0.03	.096
	Gender	0.06	−0.06 to 0.18	.311
	Age	−0.01	−0.01 to 0.00	<.001***
	Never married	0.07	−0.09 to 0.24	.376
	Education in years	−0.01	−0.02 to 0.01	.393
	Employed	−0.18	−0.32 to −0.04	.015*
	Unemployed and looking for job	0.58	0.25 to 0.92	.001***
	Permanently sick or disabled	0.23	−0.28 to 0.74	.372
	Doing housework, looking after the home, children, or other people	−0.41	−0.74 to −0.07	.017*
	Household income	−0.02	−0.04 to 0.01	.220
	Partner in same household	−0.11	−0.26 to 0.03	.132
	Urban (city, town)	0.09	−0.03 to 0.22	.140

Note: CI: 95% confidential interval, * indicates *p* ≤ .05, ** indicates *p* ≤ .01, *** indicates *p* ≤ .001.

#### Life satisfaction.

The amount of animal contact was not associated with life satisfaction when we controlled for age, marital status, education, employment status, partnership status, and settlement type (see Table 6). However, a higher amount of regular contact with other species was significantly associated with greater life satisfaction. The number of animal species, other animal species, attachment to the animal, and socio-emotional support from the animal were not included after the lasso selection.

#### Psychological well-being.

A higher amount of animal contact was tendentially associated with higher psychological well-being when we controlled for gender, age, marital status, education, employment status, household income, partnership status, and settlement type (see Table 6). Higher socio-emotional support from the animal was tendentially slightly associated with lower psychological well-being. Moreover, more regular contact with an animal kept in a terrarium or aquarium was significantly associated with lower psychological well-being. Regular contact with a dog or with a small mammal or bird was not associated with psychological well-being. Other animal species, the number of animal species, and attachment to the animal were not included after the lasso selection.

Taken together, these findings show that health status or healthcare use was not associated with attachment to animals, socio-emotional support from animal, amount of animal contact or animal species. However, a higher amount of animal contact was significantly associated with slightly more unhealthy behaviour. Higher life satisfaction was significantly associated with a higher amount of regular contact with other species but not with other factors. Higher socio-emotional support from the animal was tendentially associated with a slightly higher loneliness and with lower psychological well-being. Compared to other outcomes, psychological well-being was associated with the most factors. Higher psychological well-being was tendentially associated with a higher amount of animal contact. Lower psychological well-being was tendentially associated with higher socio-emotional support from the animal, and significantly associated with more regular contact with an animal kept in a terrarium or aquarium.

### Animal species and the amount of animal contact related to the attachment to the animal and the perceived socio-emotional support from the animal

#### Attachment to animal.

The attachment strength to the closest animal was significantly associated with the amount of animal contact: more time spent with the animal was associated with stronger attachment (see Table 7). Whether the animal lived in the household or not was significantly associated with the attachment to the animal: higher attachment was reported for animals living in the household. Women differed significantly from men, with women indicating higher attachment (women [*M* = 12.93, *SD* = 5.43], men [*M* = 10.73, *SD* = 5.77]). And the number of people in the household was significantly associated with attachment to the animal: more people in a household were associated with lower attachment to the animal. Moreover, individuals who had never been married reported a higher attachment to the animal, whereas people who were retired reported a lower attachment to the animal. Further, participants indicated significantly stronger attachment to the animal if they lived with a cat or a dog in the same household, as well as when they had regular contact with a dog. Among participants with regular animal contact, attachment to the animal was significantly lower for farm animals and for animals kept in a terrarium or aquarium (see Table 7).

**Table 7:** Animal species and the amount of animal contact related to the attachment to the animal and the perceived socio-emotional support from the animal.

Outcome	Parameter	Coefficient	95% CI	*p* value
Attachment to animal	Amount of animal contact	1.57	1.35 to 1.79	<.001***
	Animal closest to person lives in household or not	2.24	1.35 to 3.14	<.001***
	Cat in household	1.39	0.66 to 2.13	<.001***
	Dog in household	1.82	1.04 to 2.61	<.001***
	Animal kept in enclosure that can be touched (small mammal, bird) in household	−0.44	−1.38 to 0.50	.363
	Regular contact with cat	0.49	−0.15 to 1.12	.133
	Regular contact with dog	1.22	0.61 to 1.83	<.001***
	Regular contact with animal kept in enclosure that can be touched (small mammal, bird)	−0.80	−1.87 to 0.27	.143
	Regular contact with animal kept in terrarium or aquarium (fish, reptile, insect, amphibian, gastropod)	−1.17	−2.42 to 0.08	.067
	Regular contact with farm animal (cow, sheep, goat, chicken) or equine (horse, pony, donkey)	−1.00	−1.98 to −0.02	.045*
	Gender	1.55	1.01 to 2.10	<.001***
	Age	0.01	−0.02 to 0.04	.525
	Never married	0.77	0.02 to 1.53	.045*
	Education in years	−0.03	−0.10 to 0.05	.488
	Retired	−0.95	−1.86 to −0.04	.040*
	Partner in different household	0.38	−0.65 to 1.40	.471
	Number of people in household	−0.24	−0.43 to −0.04	.016*
	Urban (city, town)	−0.20	−0.80 to 0.40	.511
Socio-emotional support from animal	Amount of animal contact	1.05	0.88 to 1.22	<.001***
	Animal closest to person lives in household or not	1.67	1.02 to 2.32	<.001***
	Dog in household	0.87	0.34 to 1.40	.001***
	Animal kept in enclosure that can be touched (small mammal, bird) in household	−0.69	−1.40 to 0.01	.053
	Animal kept in terrarium or aquarium (fish, reptile, insect, amphibian, gastropod) in household	−0.48	−1.20 to 0.25	.197
	Regular contact with cat	0.39	−0.10 to 0.88	.119
	Regular contact with dog	0.81	0.36 to 1.27	<.001***
	Regular contact with animal kept in terrarium or aquarium (fish, reptile, insect, amphibian, gastropod)	−0.86	−1.73 to 0.06	.066
	Regular contact with other species	−0.33	−1.06 to 0.41	.381
	Gender	1.22	0.81 to 1.64	<.001***
	Age	−0.01	−0.02 to 0.01	.446
	Never married	0.56	0.00 to 1.13	.049*
	Education in years	−0.05	−0.11 to 0.01	.085
	Number of people in household	−0.10	−0.25 to 0.05	.180

Note: CI: 95% confidential interval, * indicates *p* ≤ .05, ** indicates *p* ≤ .01, *** indicates *p* ≤ .001.

#### Socio-emotional support from animal.

The amount of socio-emotional support from the closest animal was significantly associated with the amount of animal contact: more time spent with the animal was associated with more perceived socio-emotional support (see Table 7). Whether the animal lived in the household or not was also significant: animals living in the household were associated with higher socio-emotional support. Moreover, females and males differed significantly. Women indicated higher socio-emotional support from the animal than men (women [*M* = 7.99, *SD* = 3.93], men [*M* = 6.27, *SD* = 4.05]). Individuals who had never been married reported higher levels of socio-emotional support from the animal, while the duration of education only showed a marginally negative association with the perceived socio-emotional support from the animal. Furthermore, people with dogs living in the household reported higher levels of socio-emotional support, with a similar trend for those living with small mammals or birds. Among participants with regular animal contact outside the household, dogs were associated with higher socio-emotional support, whereas we only observed a tendency for animals kept in a terrarium or aquarium (see Table 7).

Taken together, these findings show that the attachment to the animal and the socio-emotional support from the animal are associated with several characteristics of the owner, such as gender or whether they were married, of the animal species, and of the human-animal relationship, such as the amount of animal contact or if the animal lives in the household or not.

## Discussion

In this representative, cross-sectional study, we investigated differences in health, human social support, life satisfaction, loneliness, and psychological well-being by pet ownership and regular animal contact status. We explored whether these outcomes are associated with attachment to the animal, perceived socio-emotional support from the animal, the animal species, and the amount of animal contact and whether the animal species and the amount of animal contact relate to the attachment to the animal and the perceived socio-emotional support from the animal. We found that people with pets reported a lower health status than people without pets when we controlled for relevant demographics. Moreover, we found that people with pets reported a slightly higher frequency of unhealthy behaviour (operationalised as the number of smoked cigarettes per day) compared to people without pets. Healthcare use (operationalised as the number of visits to a physician), loneliness, life satisfaction, psychological well-being, and the amount of human social support did not differ between people with and without pets. People with regular animal contact did not differ from people without regular animal contact in their self-reported health status, health behaviour, healthcare use, loneliness, life satisfaction, psychological well-being, and human social support.

### The absence of the “pet effect”

The absence of associations between pet ownership and healthcare use, loneliness, life satisfaction, psychological well-being, or the amount of human social support is in line with several studies that have also documented no association between pet ownership and human health and well-being [7,36,45,55–74,76,125]. At the same time, we could not confirm previous studies documenting better health among pet owners compared to non-pet owners [29–32]. Also, our results contrast with previous research reporting better well-being among pet owners on comparable self-reported measures, such as loneliness, psychological well-being, and life satisfaction [7,18,29,39,41,43–50,53–55,102,103]. This discrepancy between our findings and previous research reporting positive associations between pet ownership and health and well-being may partly reflect socio-demographic selection processes rather than universal effects of pet ownership itself. In contrast to many previous studies, our analyses were based on a representative sample, and we controlled for the socio-demographic factors of gender, age, marital status, education, employment status, household income, partnership status, the number of people in the household, and settlement type. Some of these variables have been found to explain differences between pet owners and non-pet owners [7,89–91]. In our sample, pet owners and individuals with regular animal contact were younger, more likely to be female, more likely to be employed or in education, and more likely to live in larger households and in rural areas compared to non-pet owners and individuals without regular animal contact. Pet owners and individuals with regular animal contact also reported lower levels of higher education and were less likely to be retired.

These sample differences align with previous population-based studies reporting that American pet owners were more likely to be younger, to be single females, to live in homes in more rural areas, and to belong to households where everyone is employed full-time [7,79]. Fraser and colleagues [79], for example, observed that pet owners were more likely to have a lower mean level of education. However, our results contrast with the finding that pet owners were more likely to be married [7,47] and partnered [79]. We assume that while pet and non-pet owners seem to differ in demographics generally, the specific characteristics might highly depend on local and socio-cultural factors, including culturally shaped roles and functions of animals, such as companionship, protection, working purposes, or urban lifestyle and aesthetic practices.

Interestingly, even when controlling for relevant socio-demographic variables, we found an association between pet ownership and lower health status, as well as slightly unhealthier behaviour. Although both effects were small, they are consistent with previous studies reporting negative or mixed associations between pet ownership and health and well-being outcomes [71,77,79–81,85]. At the same time, the observed difference in smoking behaviour was minimal, amounting to only 0.19 additional cigarettes per day on average. The effect on unhealthy behaviour must be interpreted cautiously: If this is calculated over the period of a year, this makes a difference of 70 cigarettes, but such a small difference may not reflect a meaningful lifestyle disparity. We hypothesize that this negative association could reflect a selection effect. Individuals with pre-existing health issues or higher stress levels, which are correlated with smoking [131], might be more likely to adopt a pet for comfort or companionship as a compensatory mechanism [89].

More broadly, our findings highlight the difficulty of disentangling the causal effects of pet ownership from pre-existing differences between pet owners and non-pet owners. It is possible that pet ownership leads to a lower health status and slightly more unhealthy behaviour. Another possibility is that unhealthier people are more likely to acquire pets as a compensatory mechanism and that the results can be explained by a selection effect, even though the literature largely argues that healthier people are more likely to acquire pets. Previous research has similarly demonstrated how strongly analytical approaches can influence observed “pet effects.” For example, Gmeiner and Gschwandtner [47] found that when comparing the raw data for pet carers and non-pet carers, the life satisfaction of pet carers appeared to be lower on average. However, when they used an instrumental variable approach, caring for a pet increased life satisfaction by 50% [47].

### The role of the characteristics of the human-animal relationship

In addition to pet ownership, we also investigated regular animal contact and found that regular animal contact alone was not associated with health, loneliness, life satisfaction, psychological well-being, or the amount of human social support. Similarly, we also found that attachment to the closest animal was not associated with health, loneliness, life satisfaction, and psychological well-being. However, higher socio-emotional support from the animal was significantly associated with slightly higher loneliness and tendentially slightly associated with lower psychological well-being. The amount of animal contact was significantly associated with less healthy behaviour (a slightly higher number of cigarettes smoked per day) and tendentially associated with higher psychological well-being, while it was not associated with health status, healthcare use, loneliness, or life satisfaction. Our findings suggest that the functional quality of the relationship may be more relevant than ownership status or attachment strength alone.

Our results further indicate that the quality of human–animal relationships is shaped by interaction patterns and social context. The fact that attachment to the animal was not associated is surprising, as we hypothesised that the lack of a general association between pet ownership and health and well-being could be explained by the individual relationship characteristics, such as the strength of the attachment to the animal [63,95]. Previous research has shown that people with lower social support and higher loneliness, stress, anxiety, or depression indicated a stronger attachment to their pets [57,97], suggesting that pet ownership might be particularly beneficial for vulnerable individuals. At the same time, it has been suggested that a very strong attachment to the animal may be detrimental to health, while no attachment may yield no health benefits and medium attachment optimal benefits [95].

We were nevertheless able to show that the quality of the human–animal relationship depends on several relational and socio-demographic factors. Stronger attachment and higher socio-emotional support were associated with spending more time with the animal and living in the same household as the animal, suggesting that relationship closeness may depend on the frequency and intimacy of interaction. Moreover, women and individuals who had never been married reported stronger attachment, whereas larger household size and retirement status were associated with lower attachment to the animal. The finding that women reported higher relationship quality with their pets is consistent with previous research, showing that women tend to report higher levels of positive behaviour and attitudes toward animals [132] and form a stronger attachment to pets [133]. However, within-sex variation was much higher than between-sex variation [132], and an interaction between gender and age has been observed in a Chinese population [134]. In a population of Swiss pet owners aged 55 years and older, women reported a stronger attachment to their animals than men and more frequently reported that they love their pets, that they consider them as friends, and that they enjoy having them around [135].

Regarding the animal species, the species of the pet in the household was not associated with health, loneliness, life satisfaction, or psychological well-being, which contrasts with previous research, suggesting species-specific effects on mental health and cognitive functioning [38,84]. However, relationship quality appeared to differ across animal species. Participants mostly indicated a high relationship quality with dogs. In fact, a dog or a cat living in the same household, or a dog with whom participants had regular contact, was associated with stronger attachment. Weaker attachment was associated with a farm animal or an animal kept in a terrarium or aquarium that participants had regular contact with. Similarly, both a dog living in the household and a dog with whom people had regular contact were associated with more socio-emotional support from the animal. Small mammals or birds living in the household were likewise associated with higher socio-emotional support.

### Conceptualizing underlying mechanisms

Our findings further suggest that potential associations between human–animal relationships and well-being may depend on species-specific interaction styles and the psychological mechanisms they engage. In particular, more regular contact with other species was significantly associated with more life satisfaction, and more regular contact with an animal kept in a terrarium or aquarium was associated with lower psychological well-being. The finding regarding animals kept in a terrarium or aquarium raises important questions about the mechanisms underlying species-specific human-animal interactions. From an attachment and social support perspective, the benefits of animal contact are often attributed to social interaction, emotional reciprocity, and perceived responsiveness [136], which are more characteristic of socially interactive mammals than of animals typically kept in terrariums or aquariums. Consequently, contact with less interactive species may not engage the same attachment-related or social buffering mechanisms associated with psychological well-being. This aligns with developmental findings showing that children’s empathy toward animals tends to decrease with increasing phylogenetic distance [137], suggesting that social closeness and perceived similarity may shape emotional responses to other species.

An alternative explanation for the observed association may reflect selection effects: individuals who prefer contact with non-interactive animals may differ systematically in personality traits, social needs, or lifestyle factors that themselves might relate to lower well-being. Whereas keeping dogs or cats may serve compensatory social or emotional functions, keeping terrarium or aquarium animals may primarily fulfil other motives (e.g., fascination with exotic species, aesthetic appreciation, or a preference for low-maintenance companionship) [138], which may not offer the same socio-emotional benefits. Nevertheless, a survey of reptile owners found that an emotional attachment toward reptiles was within the range of those observed in traditional pets [139]. Together, our findings suggest that the associations between human–animal relationships and health and well-being depend on complex interactions among characteristics of the owner, the animal and the human-animal relationship, such as time spent with the animal, animal species, perceived social responsiveness, relationship quality, and the owner’s emotional needs. This may help explain inconsistencies in the literature regarding the association between pet ownership and human health, and underscores the importance of conceptualizing pet ownership as a multidimensional and context-dependent phenomenon rather than a uniform construct.

### Limitations, strengths, and future directions

Several limitations of this study should be considered. First, due to the cross-sectional design of this study, it was only possible to identify associations of pet ownership and regular animal contact with health and well-being measures. Causal conclusions could not and cannot be made. The observed associations may reflect causal effects, selection processes, or compensatory mechanisms, whereby individuals with specific psychological or social characteristics are more likely to acquire pets or seek animal contact. Longitudinal and causal modelling approaches are therefore needed to better disentangle these mechanisms and clarify whether pet ownership and animal contact influence health and well-being, or whether pre-existing health characteristics influence the likelihood of acquiring pets. Moreover, caution should be taken in generalizing these results, as our analyses were exploratory, with several tested hypotheses and no correction for alpha error. Our results should be replicated and extended with other samples.

Second, we used self-reported measures for health and well-being, and each outcome was only operationalised by one item. This could lead to different results compared to objective measures of health. We acknowledge the ongoing debate regarding the reliability and validity of single-item measures [140] for assessing complex psychological constructs, such as loneliness and well-being. While some studies suggest that single-item measures have comparable validity to multiple-item scales [141,142] and may be suitable for assessing well-being [143], the limitations of single-item measures must also be considered. It is therefore possible that our measurement approach was not sufficiently sensitive to detect important differences. The lasso procedure reduces overfitting by avoiding overly complex models. However, given the number of variables, there is no guarantee that overfitting will be eliminated and – as always with variable selection – coefficients should be interpreted with care. Different items operationalizing the same construct might explain the heterogeneity in the results of the existing literature on the effects of pet ownership on health and well-being. We used items from validated scales to measure attachment to the animal and socio-emotional support from the animal, but socio-emotional support was operationalised by only two items, and attachment to the animal by only three items. Also, the amount of animal contact was measured by only one item. As we have replicated the finding that demographics seem to be relevant in investigations of the effect of pet ownership and regular animal contact on health and well-being, it must be noted that the data came from a Swiss population and that these results are not generalizable to other communities.

Several limitations also relate to the operationalization of animal species. To achieve more balanced category distributions, we aggregated certain animal species (e.g., small mammals and birds, as well as farm animals and equines) into broader categories. However, interactions with horses, for example, can differ substantially from interactions with other farm animals, which may have contributed to the null findings for species effects, despite controlling for time spent with the animal and attachment to the animal. Moreover, some less common species were represented by relatively small subsamples, and we combined them to ensure a more even distribution among species categories, limiting the generalisability of species-specific findings. Future research should therefore investigate larger and more diverse samples that allow a more differentiated analysis of species effects and interaction styles. Clarifying whether regular contact with animals outside one’s household is comparable to living with a pet also warrants further investigation. At the same time, the present study has several important strengths. We used a representative sample and included animal-related questions within a survey that people did not expect to ask about pets. This reduced the potential bias of investigating an animal-friendly population. Comparable surveys have usually only asked about the ownership of cats or dogs and have neglected other species [30], despite research that has suggested that insects and birds might also affect perceived companionship, social support, and health [45,144]. Moreover, we extended the investigation to people who have regular contact with animals outside their household and accounted for the relationship quality with the animal. To our knowledge, this was also among the first studies to combine measures of relationship characteristics, including attachment and perceived socio-emotional support from the animal, with indicators of human health and well-being in a representative population sample. This approach provides deeper insights into how different factors of pet ownership and animal contact might be associated with human health.

More broadly, our findings suggest that research on human–animal relationships should move beyond treating pet ownership as a simple binary variable. The associations between animal contact and health and well-being are complex. Future research should therefore investigate how humans interact with animals, what functions these relationships fulfil, and under which conditions they may contribute to health and well-being. For example, it might be important to include the type of interaction with the animal, such as playing, caretaking, walking, or cuddling, and pet ownership history, the date of pet adoption, the age, health, and breed of the pet, and attitudes towards animals. Longitudinal studies are needed to clarify whether the associations found here represent causal effects or selection processes. Moreover, mixed-methods approaches might enhance methodological robustness. To reliably assess the effects of different predictors, such as the animal species, and to control for variables like demographics, large datasets are needed to better account for specific effects of less prominent species. Moreover, objective health measures validated measures for well-being, attachment to the animal, and socio-emotional support from the animal should be included. Investigating these mechanisms across different cultural and social contexts may help explain the inconsistent findings regarding the associations between pet ownership and human health and well-being, and may contribute to a more theoretically integrated understanding of human–animal relationships.

### Conclusion

We found little evidence for a general association between pet ownership and human health and well-being. While pet owners reported a slightly lower health status and slightly more unhealthy behaviour, no differences emerged for healthcare use, loneliness, life satisfaction, psychological well-being, or human social support. Similarly, regular animal contact alone was not associated with health and well-being outcomes. However, relationship-related factors, including socio-emotional support from the animal and the amount of contact with the animal, were sometimes associated with human well-being. Moreover, the quality of the human–animal relationship depends on different factors. Stronger Attachment to the animal and socio-emotional support from the animal were higher in women, in people living with the animal, in people spending more time with the animal, and in people interacting with a dog.

Together, these findings suggest that associations between pet ownership and human health and well-being cannot be explained by pet ownership status alone, but depend on interactions between the characteristics of the owner, the animal and the human–animal relationship itself. Our study therefore highlights that pet ownership is a complex and heterogeneous construct that should be conceptualised multidimensionally to better understand its possible positive or negative associations with human health and well-being.

## Supporting information

S1 File Supporting information. (PDF)
