# Supplementary material for: Factors of pet ownership associated with human health: A representative cross-sectional survey of Switzerland
Source: PLoS One. 2026 Jul 29;21(7):e0352778. doi: 10.1371/journal.pone.0352778 (PMC13419196; doi:10.1371/journal.pone.0352778)
Supplement: S1 File — (PDF) [file pone.0352778.s001.pdf]

S1 Supporting information

Here, all prespecified models with the respective variables and the reduction process through the lasso method to select the final linear models (lm) are shown. "x" indicates variable included in the model.

Question 1. Differences in health status, health behaviour, healthcare use, psychological well-being, life satisfaction, loneliness, and human social support by pet ownership and regular animal contact status

S1 Table. Model 1.1: Health status.

Variable name	Variable description	Included in lasso	Included in lm
pet_now_bi	Animal in household	x	x
rc_contact_bi	Regular contact to animal	x	x
as.numeric(lonel2_n)	Loneliness	x	x
as.numeric(wellbe3_n)	Well-being	x	x
as.numeric(socsup1_n)	Human social support	x	x
sex_n	Gender	x	x
age	Age	x	x
civil_never	Never married	x	x
civil_married	Married	x	
civil_separated_a	Separated (incl. divorced and widowed)	x	
edu_j	Education in years	x	
work1_paid	In paid work	x	x
work1_unemployed	Unemployed and looking for a job	x	x
work1_education	In education (unpaid)	x	
work1_apprentice	Apprentice or trainee	x	
work1_sick	Permanently sick or disabled	x	x
work1_retired	Retired	x	x
work1_housework	Doing housework, looking after the home, children or other persons	x	x
work1_military	In compulsory military / community service	x	
as.numeric(inc1_n)	Household income	x	x
home1_no	No partner	x	x
home1_y_same	Partner in same household	x	
home1_y_not	Partner in different household	x	x
home2_n	Number of persons in household	x	x
home3_rural_a	Rural (country and village)	x	
home3_suburbs	Suburban	x	x
home3_urban_a	Urban (city and town)	x	x

S2 Table. Model 1.2: Health behaviour.

**Variable name**	**Variable description**	**Included in lasso**	**Included in lm**
pet_now_bi	Animal in household	x	x
rc_contact_bi	Regular contact to animal	x	x
as.numeric(lonel2_n)	Loneliness	x	x
as.numeric(wellbe3_n)	Well-being	x	x
as.numeric(socsup1_n)	Human social support	x	x
sex_n	Gender	x	x
age	Age	x	
civil_never	Never married	x	
civil_married	Married	x	x
civil_separated_a	Separated (incl. divorced and widowed)	x	x
edu_j	Education in years	x	x
work1_paid	In paid work	x	x
work1_unemployed	Unemployed and looking for a job	x	x
work1_education	In education (unpaid)	x	x
work1_apprentice	Apprentice or trainee	x	x
work1_sick	Permanently sick or disabled	x	x
work1_retired	Retired	x	x
work1_housework	Doing housework, looking after the home, children or other persons	x	x
work1_military	In compulsory military / community service	x	
as.numeric(inc1_n)	Household income	x	x
home1_no	No partner	x	
home1_y_same	Partner in same household	x	
home1_y_not	Partner in different household	x	x
home2_n	Number of persons in household	x	x
home3_rural_a	Rural (country and village)	x	x
home3_suburbs	Suburban	x	x
home3_urban_a	Urban (city and town)	x	

S3 Table. Model 1.3: Health care use.

Variable name	Variable description	Included in lasso	Included in lm
pet_now_bi	Animal in household	x	x
rc_contact_bi	Regular contact to animal	x	x
as.numeric(lonel2_n)	Loneliness	x	
as.numeric(wellbe3_n)	Well-being	x	x
as.numeric(socsup1_n)	Human social support	x	
sex_n	Gender	x	
age	Age	x	x
civil_never	Never married	x	
civil_married	Married	x	
civil_separated_a	Separated (incl. divorced and widowed)	x	x
edu_j	Education in years	x	x
work1_paid	In paid work	x	x
work1_unemployed	Unemployed and looking for a job	x	x
work1_education	In education (unpaid)	x	x
work1_apprentice	Apprentice or trainee	x	x
work1_sick	Permanently sick or disabled	x	x
work1_retired	Retired	x	x
work1_housework	Doing housework, looking after the home, children or other persons	x	x
work1_military	In compulsory military / community service	x	
as.numeric(inc1_n)	Household income	x	
home1_no	No partner	x	x
home1_y_same	Partner in same household	x	
home1_y_not	Partner in different household	x	
home2_n	Number of persons in household	x	
home3_rural_a	Rural (country and village)	x	x
home3_suburbs	Suburban	x	
home3_urban_a	Urban (city and town)	x	x

S4 Table. Model 2: Loneliness.

Variable name	Variable description	Included in lasso	Included in lm
pet_now_bi	Animal in household	x	x
rc_contact_bi	Regular contact to animal	x	x
as.numeric(socsup1_n)	Human social support	x	x
sex_n	Gender	x	
age	Age	x	x
civil_never	Never married	x	
civil_married	Married	x	
civil_separated_a	Separated (incl. divorced and widowed)	x	
edu_j	Education in years	x	x
work1_paid	In paid work	x	x
work1_unemployed	Unemployed and looking for a job	x	x
work1_education	In education (unpaid)	x	x
work1_apprentice	Apprentice or trainee	x	
work1_sick	Permanently sick or disabled	x	
work1_retired	Retired	x	
work1_housework	Doing housework, looking after the home, children or other persons	x	
work1_military	In compulsory military / community service	x	
as.numeric(inc1_n)	Household income	x	
home1_no	No partner	x	
home1_y_same	Partner in same household	x	
home1_y_not	Partner in different household	x	
home2_n	Number of persons in household	x	x
home3_rural_a	Rural (country and village)	x	x
home3_suburbs	Suburban	x	x
home3_urban_a	Urban (city and town)	x	

S5 Table. Model 3: Life satisfaction.

Variable name	Variable description	Included in lasso	Included in lm
pet_now_bi	Animal in household	x	x
rc_contact_bi	Regular contact to animal	x	x
as.numeric(lonel2_n)	Loneliness	x	x
as.numeric(socsup1_n)	Human social support	x	
as.numeric(healths1_n)	Health status	x	x
as.numeric(healthb2_n)	Health behavior	x	
as.numeric(healthb0_n)	Health care use	x	
sex_n	Gender	x	
age	Age	x	x
civil_never	Never married	x	x
civil_married	Married	x	
civil_separated_a	Separated (incl. divorced and widowed)	x	
edu_j	Education in years	x	x
work1_paid	In paid work	x	x
work1_unemployed	Unemployed and looking for a job	x	x
work1_education	In education (unpaid)	x	
work1_apprentice	Apprentice or trainee	x	
work1_sick	Permanently sick or disabled	x	x
work1_retired	Retired	x	
work1_housework	Doing housework, looking after the home, children or other persons	x	
work1_military	In compulsory military / community service	x	
as.numeric(inc1_n)	Household income	x	
home1_no	No partner	x	
home1_y_same	Partner in same household	x	x
home1_y_not	Partner in different household	x	
home2_n	Number of persons in household	x	
home3_rural_a	Rural (country and village)	x	x
home3_suburbs	Suburban	x	x
home3_urban_a	Urban (city and town)	x	

S6 Table. Model 4: Psychological well-being.

Variable name	Variable description	Included in lasso	Included in lm
pet_now_bi	Animal in household	x	x
rc_contact_bi	Regular contact to animal	x	x
as.numeric(lonel2_n)	Loneliness	x	x
as.numeric(socsup1_n)	Human social support	x	x
sex_n	Gender	x	x
age	Age	x	x
civil_never	Never married	x	x
civil_married	Married	x	
civil_separated_a	Separated (incl. divorced and widowed)	x	
edu_j	Education in years	x	x
work1_paid	In paid work	x	x
work1_unemployed	Unemployed and looking for a job	x	x
work1_education	In education (unpaid)	x	
work1_apprentice	Apprentice or trainee	x	
work1_sick	Permanently sick or disabled	x	x
work1_retired	Retired	x	
work1_housework	Doing housework, looking after the home, children or other persons	x	x
work1_military	In compulsory military / community service	x	
as.numeric(inc1_n)	Household income	x	x
home1_no	No partner	x	x
home1_y_same	Partner in same household	x	x
home1_y_not	Partner in different household	x	
home2_n	Number of persons in household	x	x
home3_rural_a	Rural (country and village)	x	
home3_suburbs	Suburban	x	x
home3_urban_a	Urban (city and town)	x	x

S7 Table. Model 5: Socioemotional support.

Variable name	Variable description	Included in lasso	Included in lm
pet_now_bi	Animal in household	x	x
rc_contact_bi	Regular contact to animal	x	x
sex_n	Gender	x	x
age	Age	x	x
civil_never	Never married	x	
civil_married	Married	x	
civil_separated_a	Separated (incl. divorced and widowed)	x	x
edu_j	Education in years	x	
work1_paid	In paid work	x	x
work1_unemployed	Unemployed and looking for a job	x	x
work1_education	In education (unpaid)	x	x
work1_apprentice	Apprentice or trainee	x	x
work1_sick	Permanently sick or disabled	x	x
work1_retired	Retired	x	x
work1_housework	Doing housework, looking after the home, children or other persons	x	x
work1_military	In compulsory military / community service	x	
as.numeric(inc1_n)	Household income	x	x
home1_no	No partner	x	x
home1_y_same	Partner in same household	x	x
home1_y_not	Partner in different household	x	
home2_n	Number of persons in household	x	
home3_rural_a	Rural (country and village)	x	x
home3_suburbs	Suburban	x	x
home3_urban_a	Urban (city and town)	x	

Question 2. Attachment to animals, socioemotional support from animals, amount of animal contact and animal species related to health, loneliness, life satisfaction and psychological well-being

S8 Table. Model 6.1: Health status.

Variable name	Variable description	Included in lasso	Included in lm
as.numeric(lonel2_n)	Loneliness	x	x
as.numeric(wellbe3_n)	Well-being	x	x
as.numeric(socsup1_n)	Human social support	x	x
pet_now_bi	Animal in household	x	x
rc_contact_bi	Regular contact to animal	x	x
as.numeric(spendtime_n)	Amount of spent time	x	x
pet_choice	Number of pet species	x	
pet_cat_a2	Cat in household	x	
pet_dog_a2	Dog in household	x	
pet_cage_a2	Animal kept in an enclosure who can be touched (small mammal and bird) in household	x	x
pet_TA_a2	Animal kept in a terrarium or aquarium (fish, reptile, insect, amphibian or gastropod) in household	x	
pet_farm_a2	Farm animals (cows, sheeps, goats, chickens) and equines (horses, ponies, donkeys) in household	x	x
rc_cat_a2	Cat regular contact	x	x
rc_dog_a2	Dog regular contact	x	
rc_cage_a2	Regular contact with animal kept in an enclosure who can be touched (small mammal and bird)	x	
rc_TA_a2	Regular contact with animal kept in a terrarium or aquarium (fish, reptile, insect, amphibian or gastropod)	x	x
rc_farm_a2	Regular contact with farm animals (cows, sheeps, goats, chickens) and equines (horses, ponies, donkeys)	x	
rc_other_a2	Regular contact with other species	x	
attach_score	Attachment to animal	x	x
a_socsup_score	Socioemotional support of animal	x	
sex_n	Gender	x	x
age	Age	x	x
civil_never	Never married	x	x
civil_married	Married	x	
civil_separated_a	Separated (incl. divorced and widowed)	x	
edu_j	Education in years	x	
work1_paid	In paid work	x	

work1_unemployed	Unemployed and looking for a job	x	x
work1_education	In education (unpaid)	x	x
work1_apprentice	Apprentice or trainee	x	
work1_sick	Permanently sick or disabled	x	x
work1_retired	Retired	x	
work1_housework	Doing housework, looking after the home, children or other persons	x	
work1_military	In compulsory military / community service	x	
as.numeric(inc1_n)	Household income	x	x
home1_no	No partner	x	x
home1_y_same	Partner in same household	x	
home1_y_not	Partner in different household	x	
home2_n	Number of persons in household	x	x
home3_rural_a	Rural (country and village)	x	
home3_suburbs	Suburban	x	x
home3_urban_a	Urban (city and town)	x	x

S9 Table. Model 6.2: Health behaviour.

Variable name	Variable description	Included in lasso	Included in lm
as.numeric(lonel2_n)	Loneliness	x	
as.numeric(wellbe3_n)	Well-being	x	
as.numeric(socsup1_n)	Human social support	x	x
pet_now_bi	Animal in household	x	x
rc_contact_bi	Regular contact to animal	x	x
as.numeric(spendtime_n)	Amount of spent time	x	x
pet_choice	Number of pet species	x	
pet_cat_a2	Cat in household	x	
pet_dog_a2	Dog in household	x	
pet_cage_a2	Animal kept in an enclosure who can be touched (small mammal and bird) in household	x	
pet_TA_a2	Animal kept in a terrarium or aquarium (fish, reptile, insect, amphibian or gastropod) in household	x	
pet_farm_a2	Farm animals (cows, sheeps, goats, chickens) and equines (horses, ponies, donkeys) in household	x	x
rc_cat_a2	Cat regular contact	x	
rc_dog_a2	Dog regular contact	x	
rc_cage_a2	Regular contact with animal kept in an enclosure who can be touched (small mammal and bird)	x	
rc_TA_a2	Regular contact with animal kept in a terrarium or aquarium (fish, reptile, insect, amphibian or gastropod)	x	
rc_farm_a2	Regular contact with farm animals (cows, sheeps, goats, chickens) and equines (horses, ponies, donkeys)	x	x
rc_other_a2	Regular contact with other species	x	
attach_score	Attachment to animal	x	
a_socsup_score	Socioemotional support of animal	x	
sex_n	Gender	x	x
age	Age	x	
civil_never	Never married	x	
civil_married	Married	x	
civil_separated_a	Separated (incl. divorced and widowed)	x	
edu_j	Education in years	x	x
work1_paid	In paid work	x	x
work1_unemployed	Unemployed and looking for a job	x	
work1_education	In education (unpaid)	x	
work1_apprentice	Apprentice or trainee	x	
work1_sick	Permanently sick or disabled	x	x
work1_retired	Retired	x	

work1_housework	Doing housework, looking after the home, children or other persons	x	x
work1_military	In compulsory military / community service	x	
as.numeric(inc1_n)	Household income	x	x
home1_no	No partner	x	
home1_y_same	Partner in same household	x	
home1_y_not	Partner in different household	x	
home2_n	Number of persons in household	x	x
home3_rural_a	Rural (country and village)	x	
home3_suburbs	Suburban	x	
home3_urban_a	Urban (city and town)	x	

S10 Table. Model 6.3: Health care use.

Variable name	Variable description	Included in lasso	Included in lm
as.numeric(lonel2_n)	Loneliness	x	
as.numeric(wellbe3_n)	Well-being	x	x
as.numeric(socsup1_n)	Human social support	x	
pet_now_bi	Animal in household	x	x
rc_contact_bi	Regular contact to animal	x	x
as.numeric(spendtime_n)	Amount of spent time	x	x
pet_choice	Number of pet species	x	
pet_cat_a2	Cat in household	x	
pet_dog_a2	Dog in household	x	
pet_cage_a2	Animal kept in an enclosure who can be touched (small mammal and bird) in household	x	
pet_TA_a2	Animal kept in a terrarium or aquarium (fish, reptile, insect, amphibian or gastropod) in household	x	
pet_farm_a2	Farm animals (cows, sheeps, goats, chickens) and equines (horses, ponies, donkeys) in household	x	
rc_cat_a2	Cat regular contact	x	
rc_dog_a2	Dog regular contact	x	
rc_cage_a2	Regular contact with animal kept in an enclosure who can be touched (small mammal and bird)	x	
rc_TA_a2	Regular contact with animal kept in a terrarium or aquarium (fish, reptile, insect, amphibian or gastropod)	x	
rc_farm_a2	Regular contact with farm animals (cows, sheeps, goats, chickens) and equines (horses, ponies, donkeys)	x	x
rc_other_a2	Regular contact with other species	x	
attach_score	Attachment to animal	x	
a_socsup_score	Socioemotional support of animal	x	x
sex_n	Gender	x	
age	Age	x	x
civil_never	Never married	x	
civil_married	Married	x	
civil_separated_a	Separated (incl. divorced and widowed)	x	x
edu_j	Education in years	x	x
work1_paid	In paid work	x	x
work1_unemployed	Unemployed and looking for a job	x	x
work1_education	In education (unpaid)	x	x
work1_apprentice	Apprentice or trainee	x	x
work1_sick	Permanently sick or disabled	x	x
work1_retired	Retired	x	x

work1_housework	Doing housework, looking after the home, children or other persons	x	
work1_military	In compulsory military / community service	x	
as.numeric(inc1_n)	Household income	x	
home1_no	No partner	x	x
home1_y_same	Partner in same household	x	
home1_y_not	Partner in different household	x	
home2_n	Number of persons in household	x	
home3_rural_a	Rural (country and village)	x	x
home3_suburbs	Suburban	x	
home3_urban_a	Urban (city and town)	x	x

S11 Table. Model 7: Loneliness.

Variable name	Variable description	Included in lasso	Included in lm
as.numeric(socsup1_n)	Human social support	x	x
pet_now_bi	Animal in household	x	x
rc_contact_bi	Regular contact to animal	x	x
as.numeric(spendtime_n)	Amount of spent time	x	x
pet_choice	Number of pet species	x	
pet_cat_a2	Cat in household	x	
pet_dog_a2	Dog in household	x	
pet_cage_a2	Animal kept in an enclosure who can be touched (small mammal and bird) in household	x	
pet_TA_a2	Animal kept in a terrarium or aquarium (fish, reptile, insect, amphibian or gastropod) in household	x	x
pet_farm_a2	Farm animals (cows, sheeps, goats, chickens) and equines (horses, ponies, donkeys) in household	x	
rc_cat_a2	Cat regular contact	x	
rc_dog_a2	Dog regular contact	x	
rc_cage_a2	Regular contact with animal kept in an enclosure who can be touched (small mammal and bird)	x	
rc_TA_a2	Regular contact with animal kept in a terrarium or aquarium (fish, reptile, insect, amphibian or gastropod)	x	
rc_farm_a2	Regular contact with farm animals (cows, sheeps, goats, chickens) and equines (horses, ponies, donkeys)	x	
rc_other_a2	Regular contact with other species	x	
attach_score	Attachment to animal	x	
a_socsup_score	Socioemotional support of animal	x	x
sex_n	Gender	x	
age	Age	x	x
civil_never	Never married	x	
civil_married	Married	x	
civil_separated_a	Separated (incl. divorced and widowed)	x	
edu_j	Education in years	x	x
work1_paid	In paid work	x	x
work1_unemployed	Unemployed and looking for a job	x	x
work1_education	In education (unpaid)	x	x
work1_apprentice	Apprentice or trainee	x	
work1_sick	Permanently sick or disabled	x	
work1_retired	Retired	x	
work1_housework	Doing housework, looking after the home, children or other persons	x	

work1_military	In compulsory military / community service	x	
as.numeric(inc1_n)	Household income	x	
home1_no	No partner	x	
home1_y_same	Partner in same household	x	
home1_y_not	Partner in different household	x	
home2_n	Number of persons in household	x	x
home3_rural_a	Rural (country and village)	x	x
home3_suburbs	Suburban	x	x
home3_urban_a	Urban (city and town)	x	

S 12 Table. Model 8: Life satisfaction.

Variable name	Variable description	Included in lasso	Included in lm
as.numeric(lonel2_n)	Loneliness	x	x
as.numeric(socsup1_n)	Human social support	x	x
as.numeric(healths1_n)	Health status	x	
as.numeric(healthb2_n)	Health behavior	x	
as.numeric(healthb0_n)	Health care use	x	
pet_now_bi	Animal in household	x	x
rc_contact_bi	Regular contact to animal	x	x
as.numeric(spendtime_n)	Amount of spent time	x	x
pet_choice	Number of pet species	x	
pet_cat_a2	Cat in household	x	
pet_dog_a2	Dog in household	x	
pet_cage_a2	Animal kept in an enclosure who can be touched (small mammal and bird) in household	x	
pet_TA_a2	Animal kept in a terrarium or aquarium (fish, reptile, insect, amphibian or gastropod) in household	x	
pet_farm_a2	Farm animals (cows, sheeps, goats, chickens) and equines (horses, ponies, donkeys) in household	x	
rc_cat_a2	Cat regular contact	x	
rc_dog_a2	Dog regular contact	x	
rc_cage_a2	Regular contact with animal kept in an enclosure who can be touched (small mammal and bird)	x	
rc_TA_a2	Regular contact with animal kept in a terrarium or aquarium (fish, reptile, insect, amphibian or gastropod)	x	
rc_farm_a2	Regular contact with farm animals (cows, sheeps, goats, chickens) and equines (horses, ponies, donkeys)	x	
rc_other_a2	Regular contact with other species	x	x
attach_score	Attachment to animal	x	
a_socsup_score	Socioemotional support of animal	x	
sex_n	Gender	x	
age	Age	x	x
civil_never	Never married	x	x
civil_married	Married	x	
civil_separated_a	Separated (incl. divorced and widowed)	x	
edu_j	Education in years	x	x
work1_paid	In paid work	x	x
work1_unemployed	Unemployed and looking for a job	x	x
work1_education	In education (unpaid)	x	
work1_apprentice	Apprentice or trainee	x	
work1_sick	Permanently sick or disabled	x	x

work1_retired	Retired	x	
work1_housework	Doing housework, looking after the home, children or other persons	x	
work1_military	In compulsory military / community service	x	
as.numeric(inc1_n)	Household income	x	
home1_no	No partner	x	
home1_y_same	Partner in same household	x	x
home1_y_not	Partner in different household	x	
home2_n	Number of persons in household	x	
home3_rural_a	Rural (country and village)	x	x
home3_suburbs	Suburban	x	x
home3_urban_a	Urban (city and town)	x	

S13 Table. Model 9: Psychological well-being.

Variable name	Variable description	Included in lasso	Included in lm
as.numeric(lonel2_n)	Loneliness	x	x
as.numeric(socsup1_n)	Human social support	x	x
pet_now_bi	Animal in household	x	x
rc_contact_bi	Regular contact to animal	x	x
as.numeric(spendtime_n)	Amount of spent time	x	x
pet_choice	Number of pet species	x	
pet_cat_a2	Cat in household	x	
pet_dog_a2	Dog in household	x	x
pet_cage_a2	Animal kept in an enclosure who can be touched (small mammal and bird) in household	x	
pet_TA_a2	Animal kept in a terrarium or aquarium (fish, reptile, insect, amphibian or gastropod) in household	x	x
pet_farm_a2	Farm animals (cows, sheeps, goats, chickens) and equines (horses, ponies, donkeys) in household	x	x
rc_cat_a2	Cat regular contact	x	
rc_dog_a2	Dog regular contact	x	x
rc_cage_a2	Regular contact with animal kept in an enclosure who can be touched (small mammal and bird)	x	x
rc_TA_a2	Regular contact with animal kept in a terrarium or aquarium (fish, reptile, insect, amphibian or gastropod)	x	x
rc_farm_a2	Regular contact with farm animals (cows, sheeps, goats, chickens) and equines (horses, ponies, donkeys)	x	
rc_other_a2	Regular contact with other species	x	x
attach_score	Attachment to animal	x	
a_socsup_score	Socioemotional support of animal	x	x
sex_n	Gender	x	x
age	Age	x	x
civil_never	Never married	x	x
civil_married	Married	x	
civil_separated_a	Separated (incl. divorced and widowed)	x	
edu_j	Education in years	x	x
work1_paid	In paid work	x	x
work1_unemployed	Unemployed and looking for a job	x	x
work1_education	In education (unpaid)	x	
work1_apprentice	Apprentice or trainee	x	
work1_sick	Permanently sick or disabled	x	x
work1_retired	Retired	x	
work1_housework	Doing housework, looking after the home, children or other persons	x	x

work1_military	In compulsory military / community service	x	
as.numeric(inc1_n)	Household income	x	x
home1_no	No partner	x	
home1_y_same	Partner in same household	x	x
home1_y_not	Partner in different household	x	
home2_n	Number of persons in household	x	
home3_rural_a	Rural (country and village)	x	
home3_suburbs	Suburban	x	
home3_urban_a	Urban (city and town)	x	x

Question 3. Animal species and the amount of animal contact related to the attachment to the animal and the perceived socio-emotional support from the animal

S 14 Table. Model 10.1: Attachment to animal.

Variable name	Variable description	Included in lasso	Included in lm
as.numeric(spendtime_n)	Amount of spent time	x	x
whichanimal_n	Animal which is closest to person lives in household or not	x	x
as.numeric(socsup1_n)	Human social support	x	
pet_choice	Number of pet species	x	
pet_cat_a2	Cat in household	x	x
pet_dog_a2	Dog in household	x	x
pet_cage_a2	Animal kept in an enclosure who can be touched (small mammal and bird) in household	x	x
pet_TA_a2	Animal kept in a terrarium or aquarium (fish, reptile, insect, amphibian or gastropod) in household	x	
pet_farm_a2	Farm animals (cows, sheeps, goats, chickens) and equines (horses, ponies, donkeys) in household	x	
rc_cat_a2	Cat regular contact	x	x
rc_dog_a2	Dog regular contact	x	x
rc_cage_a2	Regular contact with animal kept in an enclosure who can be touched (small mammal and bird)	x	x
rc_TA_a2	Regular contact with animal kept in a terrarium or aquarium (fish, reptile, insect, amphibian or gastropod)	x	x
rc_farm_a2	Regular contact with farm animals (cows, sheeps, goats, chickens) and equines (horses, ponies, donkeys)	x	x
rc_other_a2	Regular contact with other species	x	
sex_n	Gender	x	x
age	Age	x	x
civil_never	Never married	x	x
civil_married	Married	x	
civil_separated_a	Separated (incl. divorced and widowed)	x	
edu_j	Education in years	x	x
work1_paid	In paid work	x	
work1_unemployed	Unemployed and looking for a job	x	
work1_education	In education (unpaid)	x	
work1_apprentice	Apprentice or trainee	x	
work1_sick	Permanently sick or disabled	x	
work1_retired	Retired	x	x

work1_housework	Doing housework, looking after the home, children or other persons	x	
work1_military	In compulsory military / community service	x	
as.numeric(inc1_n)	Household income	x	
home1_no	No partner	x	
home1_y_same	Partner in same household	x	
home1_y_not	Partner in different household	x	x
home2_n	Number of persons in household	x	x
home3_rural_a	Rural (country and village)	x	
home3_suburbs	Suburban	x	
home3_urban_a	Urban (city and town)	x	x

S 15 Table. Model 10.2: Socioemotional support of animal.

Variable name	Variable description	Included in lasso	Included in lm
as.numeric(spendtime_n)	Amount of spent time	x	x
whichanimal_n	Animal which is closest to person lives in household or not	x	x
as.numeric(socsup1_n)	Human social support	x	
pet_choice	Number of pet species	x	
pet_cat_a2	Cat in household	x	
pet_dog_a2	Dog in household	x	x
pet_cage_a2	Animal kept in an enclosure who can be touched (small mammal and bird) in household	x	x
pet_TA_a2	Animal kept in a terrarium or aquarium (fish, reptile, insect, amphibian or gastropod) in household	x	x
pet_farm_a2	Farm animals (cows, sheeps, goats, chickens) and equines (horses, ponies, donkeys) in household	x	
rc_cat_a2	Cat regular contact	x	x
rc_dog_a2	Dog regular contact	x	x
rc_cage_a2	Regular contact with animal kept in an enclosure who can be touched (small mammal and bird)	x	
rc_TA_a2	Regular contact with animal kept in a terrarium or aquarium (fish, reptile, insect, amphibian or gastropod)	x	x
rc_farm_a2	Regular contact with farm animals (cows, sheeps, goats, chickens) and equines (horses, ponies, donkeys)	x	
rc_other_a2	Regular contact with other species	x	x
sex_n	Gender	x	x
age	Age	x	x
civil_never	Never married	x	x
civil_married	Married	x	
civil_separated_a	Separated (incl. divorced and widowed)	x	
edu_j	Education in years	x	x
work1_paid	In paid work	x	
work1_unemployed	Unemployed and looking for a job	x	
work1_education	In education (unpaid)	x	
work1_apprentice	Apprentice or trainee	x	
work1_sick	Permanently sick or disabled	x	
work1_retired	Retired	x	
work1_housework	Doing housework, looking after the home, children or other persons	x	
work1_military	In compulsory military / community service	x	
as.numeric(inc1_n)	Household income	x	

home1_no	No partner	x	
home1_y_same	Partner in same household	x	
home1_y_not	Partner in different household	x	
home2_n	Number of persons in household	x	x
home3_rural_a	Rural (country and village)	x	
home3_suburbs	Suburban	x	
home3_urban_a	Urban (city and town)	x	
