# Supplementary material for: Factors of pet ownership associated with human health: A representative cross-sectional survey of Switzerland
Source: PLoS One. 2026 Jul 29;21(7):e0352778. doi: 10.1371/journal.pone.0352778 (PMC13419196; doi:10.1371/journal.pone.0352778)
Supplement: Attachment — Submitted filename: Point-by-point-response_Revision.docx [file pone.0352778.s004.docx]

Point-by-point-response

**Factors of pet ownership associated with human health: a representative cross-sectional survey of Switzerland**

1. While the introduction section meticulously enumerates the inconsistent findings of past research on the pet effect (some beneficial, some non-beneficial or harmful), it lacks an integrative psychological theoretical framework to explain these discrepancies. Simply attributing the results to methodological weaknesses or demographic differences is insufficient.

Thank you for this important comment. We agree that a psychological theoretical framework is essential to explain the discrepancies observed in our findings. Accordingly, we discuss several relevant frameworks such as selection processes (e.g., healthier or less healthy individuals being more likely to adopt a pet), attachment processes as potential moderators (starting at line 138), and other psychological factors such as mindfulness experienced in the presence of pets (line 156). Based on these considerations, we argue that research should account not only for sociodemographic factors but also for social support, the amount of contact with the animal, and attachment to the animal.

It is possible that these psychological theoretical frameworks were not sufficiently clear to readers. To address this, we have extended the Introduction by adding the following sentences:

*Line 128: This raises the question of whether pet ownership leads to changes in health, or whether individuals with better or poorer physical or mental health are more likely to acquire pets. In the latter case, observed associations would not reflect causal effects but rather underlying selection processes.*

*Line 141:* *The heterogeneity in results can also be explained by further psychological theoretical frameworks, such as attachment processes and social support theory. Loneliness or living alone can have a mediating role, driving another selection process* (98)*. It has been hypothesized that individuals who experience loneliness, anxiety, or depressive symptoms may be more likely to adopt a pet as a form of self-medication, indicating a compensatory mechanism* (99) *whereby, e.g., lonely individuals seek stronger emotional bonds with pets to compensate a lack of human social support.*

2. Your results indicate that higher socio-emotional support from animals is associated with higher levels of loneliness. From a psychological perspective, could this suggest a compensatory mechanism rather than a causal one? In other words, do lonely individuals seek out stronger emotional bonds with pets to compensate for a lack of human social support?

Thank you for this helpful comment. Indeed, this is a mechanism that is often hypothesized. We have already inserted this according to your previous comment (see above):

*Line 143: It has been hypothesized that individuals who experience loneliness, anxiety, or depressive symptoms may be more likely to adopt a pet as a form of self-medication, indicating a compensatory mechanism* (99) *whereby, e.g., lonely individuals seek stronger emotional bonds with pets to compensate a lack of human social support.*

3. The study finds that pet owners reported slightly lower health status and higher frequency of unhealthy behaviors like smoking. Could you elaborate more on the selection effect in your discussion? Specifically, from a health psychology standpoint, is it plausible that individuals with pre-existing health issues or higher stress levels (correlated with smoking) are more likely to acquire pets for comfort or companionship?

Thank you for your helpful comment; you are absolutely right. We have now included this point in the Discussion section, following the corresponding addition to the Introduction:

*Line 882: We hypothesize that this negative association could reflect a selection effect. Individuals with pre-existing health issues or higher stress levels, which are correlated with smoking (124), might be more likely to adopt a pet for comfort or companionship as a compensatory mechanism.*

4. I note that psychological constructs such as loneliness and psychological well-being were measured using single items. From a psychometric perspective, single-item measures often have lower reliability and validity compared to established scales. Could the limited variance captured by these single items influence the result? Please add a discussion on how this measurement limitation might affect the sensitivity of your results.

Thank you for this important comment. We agree with you that single-item measures can have weaknesses. However, they can also have strengths as outlined by the editors of the World Happiness Report 2025 who explained in their article why single-item measures of wellbeing are best (Helliwell, J.F., Layard, R., Sachs, J.D. *et al.* (2026). Why single-item measures of wellbeing are best. *Nature Human Behavior*. <https://doi.org/10.1038/s41562-026-02401-y>). But we acknowledge the debate and extended the limitations section as follows:

Line 1081: *We acknowledge the ongoing debate regarding the reliability and validity of single-item measures (132) for assessing complex psychological constructs such as loneliness and well-being. While some studies suggest that single-item measures have comparable validity to multiple-item scales (133,134) and may be suitable for assessing well-being (135), the limitations of single-item measures must also be considered. It is therefore possible that our measurement approach was not sufficiently sensitive to detect important differences.*

5. The result that pet owners smoke ‘0.19 cigarettes more per day’ is statistically significant but appears to lack practical or clinical significance. Emphasizing this as a finding of ‘unhealthy behavior’ seems overstated. The authors should provide effect sizes (e.g., Cohen's d) or interpret this magnitude with more caution, acknowledging that such a small difference may not reflect a meaningful lifestyle disparity.

Thank you for raising this important point. We agree with you that this is a very small effect. As we used the difference as effect size, we changed the wording and added “slightly” throughout the manuscript. Moreover, we extended the limitations:

*Line 725: …significantly but only slightly higher frequency of unhealthy behaviour…*

*Line 862: …reported a slightly higher frequency of unhealthy behaviour (operationalized as the number of smoked cigarettes per day)…*

*Line 878: …, apart from a slightly higher frequency of smoking cigarettes in pet owners compared to non-pet owners.*

*Line 879: The effect on unhealthy behaviour must be interpreted cautiously as the number of smoked cigarettes per day only differed by 0.19 cigarettes more per day. If this is calculated over the period of a year, this makes a difference of 70 cigarettes, but such a small difference may not reflect a meaningful lifestyle disparity.*

*Line 925: … a slightly unhealthier behaviour…*

*Line 928: … a slightly unhealthier behaviour…*

6. The aggregation of ‘Equines’ with ‘Farm animals’ and ‘Rodents’ with ‘Birds’ to ensure sample size may dilute species-specific effects. Psychologically, the interaction with a horse (often involving sport and high engagement) is vastly different from interacting with farm livestock. This limitation might explain the null results regarding species and should be acknowledged as a constraint on the study's ability to detect species-specific benefits.

Thank you for this important comment. We agree that it would have been interesting to analyze the animal species separately. However, the number of observations for some species was too small, resulting in highly uneven category distributions, which would have posed challenges for the statistical models. For example, we only had 26 animals in the category “farm animals and equines” together. We therefore aimed to define broader categories of animal species that share at least some common characteristics regarding interaction. As we controlled for the amount of time spent and attachment to the pet, some of the effects are diminished, but still, this aggregation may have led to the null results. This limitation has been acknowledged in the manuscript as follows:

*Line 1092: To achieve more balanced category distributions, we aggregated certain animal species (e.g., rodents and birds, as well as farm animals and equines) into broader categories. However, interactions with horses, for example, can differ substantially from interactions with other farm animals, which may have contributed to the null findings for species effects, despite controlling for time spent with the animal and attachment to the animal.*

7. The finding that regular contact with animals kept in terrariums/aquariums was associated with lower psychological well-being is intriguing. From an evolutionary or environmental psychology perspective, what might explain this? Does this reflect the solitary nature of such hobbies, or perhaps a different psychological motivation for keeping non-interactive pets compared to social mammals like dogs? Expanding on the psychological function of different species would add depth to your discussion on species-specific effects.

Thank you for this comment. We also find this result interesting and happily extended our discussion:

Line 961: *The finding regarding animals kept in a terrarium or aquarium raises important questions about the mechanisms underlying species-specific human-animal interactions. From an attachment and social support perspective, the benefits of animal contact are often attributed to social interaction, emotional reciprocity, and perceived responsiveness (125), which are more characteristic of socially interactive mammals than of animals typically kept in terrariums or aquariums. Consequently, contact with less interactive species may not engage the same attachment-related or social buffering mechanisms associated with psychological well-being. This aligns with developmental findings showing that children’s empathy toward animals tends to decrease with increasing phylogenetic distance (126), suggesting that social closeness and perceived similarity may shape emotional responses to other species. An alternative explanation for the observed association may reflect selection effects: individuals who prefer contact with non-interactive animals may differ systematically in personality traits, social needs, or lifestyle factors that themselves might relate to lower well-being. Whereas keeping dogs or cats may serve compensatory social or emotional functions, keeping terrarium or aquarium animals may primarily fulfil other motives (e.g., fascination with exotic species, aesthetic appreciation, or a preference for low-maintenance companionship) (127), which may not offer the same socioemotional benefits. Nevertheless, a survey on reptile owners found that an emotional attachment toward reptiles was within the range of those observed in traditional pets (128). Together, these findings underscore the importance of considering species-specific interaction styles and individual differences when evaluating well-being and pet ownership.*

8. In the Discussion, you suggest that socio-demographic differences might explain the lack of a pet effect. However, your regression models controlled for these variables (gender, age, settlement type, etc.) and still found a negative association with health status. The discussion needs to address why the negative association persists after adjustment. This suggests if there existed some other factors or unmeasured confounders, rather than just demographics.

You are correct. While our models controlled for key socio-demographic factors, the negative association between pet ownership and health status persisted. This is addressed in the discussion from line 925 on. We have extended the arguments to make clear that beyond socio-demographic factors, compensatory mechanisms and the selection effect might be relevant in explaining our results.

Line 928: *Another possibility is that unhealthier people are more likely to acquire pets as a compensatory mechanism and that the results can be explained by a selection effect, even though the literature largely argues that healthier people are more likely to acquire pets.*
