# Supplementary material for: Factors of pet ownership associated with human health: A representative cross-sectional survey of Switzerland
Source: PLoS One. 2026 Jul 29;21(7):e0352778. doi: 10.1371/journal.pone.0352778 (PMC13419196; doi:10.1371/journal.pone.0352778)
Supplement: Attachment — Submitted filename: comment.docx [file pone.0352778.s002.docx]

1. While the introduction section meticulously enumerates the inconsistent findings of past research on the pet effect (some beneficial, some non-beneficial or harmful), it lacks an integrative psychological theoretical framework to explain these discrepancies. Simply attributing the results to methodological weaknesses or demographic differences is insufficient.
2. Your results indicate that higher socio-emotional support from animals is associated with higher levels of loneliness. From a psychological perspective, could this suggest a compensatory mechanism rather than a causal one? In other words, do lonely individuals seek out stronger emotional bonds with pets to compensate for a lack of human social support?
3. The study finds that pet owners reported slightly lower health status and higher frequency of unhealthy behaviors like smoking. Could you elaborate more on the selection effect in your discussion? Specifically, from a health psychology standpoint, is it plausible that individuals with pre-existing health issues or higher stress levels (correlated with smoking) are more likely to acquire pets for comfort or companionship?
4. I note that psychological constructs such as loneliness and psychological well-being were measured using single items . From a psychometric perspective, single-item measures often have lower reliability and validity compared to established scales. Could the limited variance captured by these single items influence the result? Please add a discussion on how this measurement limitation might affect the sensitivity of your results.
5. The result that pet owners smoke ‘0.19 cigarettes more per day’ is statistically significant but appears to lack practical or clinical significance. Emphasizing this as a finding of ‘unhealthy behavior’ seems overstated. The authors should provide effect sizes (e.g., Cohen's d) or interpret this magnitude with more caution, acknowledging that such a small difference may not reflect a meaningful lifestyle disparity.
6. The aggregation of ‘Equines’ with ‘Farm animals’ and ‘Rodents’ with ‘Birds’ to ensure sample size may dilute species-specific effects. Psychologically, the interaction with a horse (often involving sport and high engagement) is vastly different from interacting with farm livestock. This limitation might explain the null results regarding species and should be acknowledged as a constraint on the study's ability to detect species-specific benefits.
7. The finding that regular contact with animals kept in terrariums/aquariums was associated with lower psychological well-being is intriguing. From an evolutionary or environmental psychology perspective, what might explain this? Does this reflect the solitary nature of such hobbies, or perhaps a different psychological motivation for keeping non-interactive pets compared to social mammals like dogs? Expanding on the psychological function of different species would add depth to your discussion on species-specific effects.
8. In the Discussion, you suggest that socio-demographic differences might explain the lack of a pet effect. However, your regression models controlled for these variables (gender, age, settlement type, etc.) and still found a negative association with health status. The discussion needs to address why the negative association persists after adjustment. This suggests if there existed some other factors or unmeasured confounders, rather than just demographics.
