# Supplementary material for: Factors of pet ownership associated with human health: A representative cross-sectional survey of Switzerland
Source: PLoS One. 2026 Jul 29;21(7):e0352778. doi: 10.1371/journal.pone.0352778 (PMC13419196; doi:10.1371/journal.pone.0352778)
Supplement: Attachment — Submitted filename: Point-by-point-response_Reviewer1_final.docx [file pone.0352778.s005.docx]

**MOSAiCH -** **Factors of pet ownership associated with human health: a representative cross-sectional survey of Switzerland**

**Point-by-point response**

# **Reviewer 1**

This study examines how pet ownership and regular animal contact status affect psychological well-being, life satisfaction, social support, loneliness, and health. My observations are given below:

(1) The study title is okay. The abstract needs some revisions. The abstract is well-structured and understandable, but it is too descriptive and doesn't concentrate on the theoretical justification or implications. The mechanical summary of statistical results does not emphasize significant levels or important patterns. The general conclusion provides little understanding of the underlying mechanisms or application.

We thank the reviewer for this thoughtful comment. We agree with you that theoretical justification is important. We thus added the following sentence to emphasize the theoretical justification, see line 21: “*It is suggested that factors related to the owner, the animal and the human–animal relationship might explain some of the previous heterogeneity in findings.”*

As there is a word limit for the abstract and the effect sizes are small, we decided to use the effect wording and not to present the significance levels within the results to avoid overinterpretation. However, if the editor would like us to add the estimates and CI’s together with the p-values, we are happy to include these also in the abstract.

We were cautious to not overinterpreting our findings and did not want to imply anything into the data that cannot be justified as assuming underlying mechanisms would be based on interpretation. However, we acknowledge the reviewer’s request and concern and added a the following text, see line 45: “*We found little evidence for a general association between pet ownership or regular animal contact and human health and well-being. However, relationship-related factors, including socio-emotional support from the animal and the amount of contact with the animal, were sometimes associated with well-being and the quality of the human–animal relationship depends on different factors such as the amount of spent time or animal species. These findings suggest that associations between pet ownership and human health and well-being depend on characteristics of the owner, the animal and the human–animal relationship itself*.”

(2) The introduction needs revisions. A thorough and well-structured review of the literature on the connection between pet ownership and human health and wellbeing is given in the introduction. It successfully demonstrates the importance of the subject and points out discrepancies in previous studies. But the main point is weakened by its excessive length and occasional repetition. Although stated, the justification for this study could be made clearer. Increased theoretical foundation and conceptual integration would improve coherence and make it clearer how this research advances our knowledge of human-animal relationships.

We thank the reviewer for this thoughtful and constructive comment. We agree that the introduction benefits from a clearer conceptual structure and stronger theoretical integration.

In response, we substantially revised and reorganized the introduction to improve coherence, reduce redundancy, and clarify the rationale and contribution of the study. First, we condensed several sections of the literature review, particularly the overview of positive effects of pet ownership, by synthesizing findings more compactly and reducing repetitive examples (see page 4-6). Second, we restructured the discussion of heterogeneity in previous findings into conceptually distinct sections addressing: (1) socio-demographic factors and selection processes, (2) attachment and social support theory, (3) animal species and interaction intensity, and (4) psychological and neurobiological mechanisms underlying human–animal relationships (see page 7-11). This restructuring was intended to strengthen the theoretical framework and improve the logical flow of the introduction.

In addition, we revised the study rationale to more explicitly emphasize that associations between pet ownership and health may depend not only on ownership status itself, but also on interacting owner-, animal-, and relationship-related factors, including socio-demographic characteristics, attachment to the animal, perceived socio-emotional support, amount of animal contact, and animal species. We also clarified that the present study advances previous research by examining these dimensions simultaneously within a nationally representative sample (see line 253).

Together, these revisions were intended to improve conceptual integration, reduce repetition, and more clearly position the study’s contribution to understanding the complexity of human–animal relationships and their associations with health and well-being.

(3) The methods section needs modifications. The methods section improves representativeness and replicability by showcasing meticulous sampling and open data collection practices using the MOSAiCH survey. However, single-item measures for complex constructs (e.g., causal inference) are limited using cross-sectional design. A. loneliness, wellbeing) could diminish validity and dependability. It runs the risk of missing conceptual variance if only highly correlated items are chosen. While the statistical rigor demonstrated by LASSO regression and imputation is praiseworthy, interpretability is weakened by overfitting and a lack of theoretical support for variable inclusion. Methodological robustness may be enhanced by longitudinal or mixed-method approaches.

Thank you for these important comments. The other reviewer has also pointed out the use of single-item measures. We agree with you that single-item measures can have weaknesses and can lead to potential missing conceptual variance. Of course, each dimension reduction approach if PCA or simply selecting one representative variable will reduce the variance of a high dimensional construct. We fear that there is not much we can do given the amount of potentially interesting variables. However, they can also have strengths as outlined by e.g. the editors of the World Happiness Report 2025 who explained in their article why single-item measures of wellbeing are best (Helliwell, J.F., Layard, R., Sachs, J.D. *et al.* (2026). Why single-item measures of wellbeing are best. *Nature Human Behavior*. <https://doi.org/10.1038/s41562-026-02401-y>). But we take the reviewers concern serious and acknowledge the debate and thus extended the limitations section as follows:

Line 1023: *We acknowledge the ongoing debate regarding the reliability and validity of single-item measures (143) for assessing complex psychological constructs such as loneliness and well-being. While some studies suggest that single-item measures have comparable validity to multiple-item scales (144,145) and may be suitable for assessing well-being (146, the limitations of single-item measures must also be considered. It is therefore possible that our measurement approach was not sufficiently sensitive to detect important differences.*

With respect to overfitting, we are a bit less worried than the reviewer. Of course, LASSO does not prevent overfitting similarly well than building and validating statistical models with different datasets, but LASSO combined with in-built cross-validation has in many simulation studies shown to substantially reduce overfitting. Anyway, we added the following sentence to the limitation section, see line 1029: *The lasso procedure reduces overfitting by avoiding overly complex models. However, given the number of variables there is no guarantee that overfitting will be eliminated and – as always with variable selection – coefficients should be interpreted with care.*

And to address the comment regarding enhancing methodological robustness by longitudinal or mixed-method approaches, we extended the suggestions for future research as follows (see line 1081): *Future research should therefore investigate how humans interact with animals, what functions these relationships fulfil, and under which conditions they may contribute to health and well-being. Longitudinal studies are needed to clarify whether the associations found here represent causal effects or selection processes. Moreover, mixed-methods approaches might enhance methodological robustness.*

(4) The results require some modifications. Although there is a lot of descriptive and inferential information in the results section, the interpretation is not clear or succinct. Even though the statistical reporting is thorough, the results mostly show weak or non-significant associations, which raises concerns about their practical applicability. Clearer integration of descriptive and inferential findings and a targeted summary emphasizing important trends would improve the results. The rationale for controlling particular covariates should be supported, and the inclusion of many variables without a clear explanation suggests overcomplexity. The results are comprehensive overall, but they are not sufficiently synthesized to allow for meaningful interpretation.

Thank you for your important comments. We address the two separate points as follows:

**A) Integration of descriptive and inferential findings, a targeted summary with important trends and synthesizing.** The reviewer states that the interpretation of the results is not clear or succinct. We acknowledge this point but decided not to add interpretations in the results section but rather interpret the findings in the discussion. However, we agree with the reviewer that the results section profited from clearer integration and synthetization. We added the following passages:

Line 545: *“The descriptive statistics shows that pet owners or people with regular animal contact were in general four years younger and were more likely to be female compared to non-pet owners and people without regular animal contact. Their marital status was comparable, while pet owners or people with regular animal contact had completed vocational training slightly more often but possessed a degree from a university or a university of applied sciences less often. Pet owners or people with regular animal contact were also more likely to be employed or to be in education and were less likely to be retired compared to non-pet owners and people without regular animal contact. The presence of a partner and household income were comparable, while pet owners or people with regular animal contact were less likely to live in a household with one or two people and more likely to live in a household with three or more people. Moreover, pet owners or people with regular animal contact were less likely to live in a city, suburb, or town and more likely lived in a village or the countryside than non-pet owners and people without regular animal contact*.”

Line 606: “*Taken together, these findings show that pet owners reported a significantly lower self-reported health status and a slightly higher frequency of unhealthy behaviour compared to people without pets. People with and without regular animal contact did not differ in any of the health or well-being outcomes*.”

Line 665: *Taken together, these findings show that health status or healthcare use was not associated with attachment to animals, socio-emotional support from animal, amount of animal contact or animal species. However, a higher amount of animal contact was significantly associated with slightly more unhealthy behaviour. Higher life satisfaction was significantly associated with higher amount of regular contact with other species but not with other factors. Higher socio-emotional support from the animal was tendentially associated with a slightly higher loneliness and with lower psychological well-being. Compared to other outcomes, psychological wellbeing was associated with the most factors. Higher psychological well-being was tendentially associated with a higher amount of animal contact. Lower psychological well-being was tendentially associated with higher socio-emotional* *support from the animal and significantly associated with more regular contact with an animal kept in a terrarium or aquarium.”*

Line 718: “*Taken together, these findings show that the attachment to the animal and the socio-emotional support from the animal are associated with several characteristics of the owner such as gender or whether they were married, of the animal species and of the human-animal relationship such as the amount of animal contact or if the animal lives in the household or not.*”

**B) Rationale for the inclusion of predictors and covariates.** A large part of the introduction functions as foundation or rationale for the inclusion of the variables and covariates that we use in our analyses. However, we agree that this can be made more explicit and added a rationale for the **predictors** in the methods section where we describe each theoretical model.

Line 451: *These predictors were chosen based on literature showing that human health highly depends on perceived loneliness, human social support and psychological well-being (94,123).*

Line 461: *The predictor was chosen based on literature showing that loneliness is often closely linked to human social support (128).*

Line 466: *Predictors were chosen because they can all be closely linked to each other, as recent studies show (129,130).*

Line 473*: The choice of the predictors was based on literature (92,131,132).*

Line 482: *The choice of predictors had the same basis as mentioned above.*

The basis for the rationale for inclusion of the **covariates** is given in the introduction. First, we introduce what socio-demographic variables are important according to the literature when it comes to physical and mental health and pet-ownership. See from line 158. Then, we explain why the intensity of the relationship and the strength of the attachment to the animal are important, as they may mediate the association between pet ownership and well-being (see line 183). Third, we introduce that animal species and patterns of interaction with the animal is an important aspect that may mediate the association between pet ownership and well-being (see from line 196).

However, we understand that it is helpful for readers to justify the inclusion of the specific covariates more clearly. To address this, we extended the following sentence:

Line 264: *To address these aims, we analysed …. and controlled for different socio-demographic factors* ***that have been identified as important in previous literature****.*

We also added the rationale for the use of age, gender, relationship status and social network, socioeconomic status, living area, educational level, employment status as socio-demographic variables in the methods section:

Line 330: *These socio-demographic variables were chosen as previous literature has identified those as important influencing factors for human physical and mental health (1,2,4,7,79,92,93).*

Line 456: *The socio-demographic variables were chosen based on previous literature identifying them as important factors for human health (1,2,4,7,79,92,93).*

(5) The discussion needs some revisions. It is thorough and well-structured, but it frequently repeats findings and is unduly descriptive rather than delving deeper into theoretical interpretation. Critical synthesis to explain discrepancies or contextual moderators, like socioeconomic factors or cultural attitudes toward pets, is lacking in this section, even though comparisons with previous research are properly cited. Although the causal ambiguity is recognized, the limitations are frequently brought up. More integrative implications for future research could be made, emphasizing theoretical or applied contributions as opposed to just methodological improvements. Clarity is generally good, but there is little analytical depth.

We thank the reviewer for this helpful comment. We substantially revised the discussion section to improve conceptual integration, analytical depth, and coherence. Specifically, we reduced redundancies and repetitive reporting of findings by combining several result sections and streamlining descriptive passages. We further restructured the discussion according to the conceptual topics introduced in the introduction, including three new sub-headings: *The absence of the “pet effect”* (from line 745), *Ther role of the characteristics of the human-animal relationship* (from line 843), and *Conceptualizing underlying mechanisms* (from line 967).

In addition, we expanded the theoretical interpretation of our findings by discussing contextual moderators and possible explanations for heterogeneity in literature, including socio-demographic factors, species-specific interaction styles, perceived social responsiveness, compensatory mechanisms, and the multidimensional nature of pet ownership. We also strengthened the conceptual synthesis throughout the discussion and conclusion by emphasizing that associations between pet ownership and health may depend on complex interactions between owner characteristics, relationship quality, animal species, and social context, rather than on pet ownership status alone.

To reduce repetition of methodological limitations within the main discussion, we moved these aspects into the separate “Limitations, strengths, and future directions” section. In this section, we additionally expanded the implications for future research beyond methodological recommendations by highlighting the importance of investigating the mechanisms, functions, and contextual conditions underlying human–animal relationships.

(6) Please modify and check reference styles as per the journal guidelines.

We thank the reviewer for pointing this out. We carefully checked and revised the reference formatting throughout the manuscript according to the PLOS One journal guidelines. The references and citations were formatted using the official PLOS One Zotero citation style available from the Zotero Style Repository (<https://www.zotero.org/styles/plos-one>), and additional manual checks were performed to ensure consistency with the journal requirements.

In addition to the points above, I would like to highlight several aspects that require attention from an editorial perspective. The limitations inherent to the cross-sectional design should be more clearly emphasized, particularly regarding the inability to establish causality and the possibility of reverse causation and selection effects. The use of single-item measures for complex constructs such as loneliness and psychological well-being should also be more critically discussed, considering potential limitations in validity and sensitivity.

Thank you for this important comment. We extended the discussion of the limitations of the cross-sectional design of the study.

*Line 1006: First, due to the cross-sectional design of this study, it was only possible to identify associations of pet ownership and regular animal contact with health and well-being measures. Causal conclusions could not and cannot be made. The observed associations may reflect causal effects, selection processes, or compensatory mechanisms, whereby individuals with specific psychological or social characteristics are more likely to acquire pets or seek animal contact. Longitudinal and causal modelling approaches are therefore needed to better disentangle these mechanisms and clarify whether pet ownership and animal contact influence health and well-being or whether pre-existing health characteristics influence the likelihood of acquiring pets.*

As mentioned above, we extended the limitations section to discuss the challenge of using single-item measures as follows:

Line 1023: *We acknowledge the ongoing debate regarding the reliability and validity of single-item measures (143) for assessing complex psychological constructs such as loneliness and well-being. While some studies suggest that single-item measures have comparable validity to multiple-item scales (144,145) and may be suitable for assessing well-being (146), the limitations of single-item measures must also be considered. It is therefore possible that our measurement approach was not sufficiently sensitive to detect important differences.*

Furthermore:

-The analytical strategy would benefit from additional clarification, including a more explicit justification for the inclusion of covariates and an assessment of potential collinearity among predictors.

Thank you for again mentioning justification for the inclusion of covariates. We have included this now in the manuscript. The covariates are a highly used and recommended set of sociodemographic variables that should be controlled for when it comes to mental and physical health. The basis of the justification is laid in the introduction, where we introduce what socio-demographic variables are important according to the literature when it comes to physical and mental health and pet-ownership. See from line 158. Then, we explain why the intensity of the relationship and the strength of the attachment to the animal are important, as they may mediate the association between pet ownership and well-being (see line 183). Third, we introduce that animal species and patterns of interaction with the animal is an important aspect that may mediate the association between pet ownership and well-being (see from line 196).

Moreover, we extended the following sentence: Line 264: *To address these aims, we analysed …. and controlled for different socio-demographic factors* ***that have been identified as important in previous literature****.*

We also added the rationale for the use of age, gender, relationship status and social network, socioeconomic status, living area, educational level, employment status as socio-demographic variables in the methods section:

Line 330: *These socio-demographic variables were chosen as previous literature has identified those as important influencing factors for human physical and mental health (1,2,4,7,79,92,93).*

Line 456: *The socio-demographic variables were chosen based on previous literature identifying them as important factors for human health (1,2,4,7,79,92,93).*

We appreciate the reviewer’s comment regarding potential multicollinearity among the variables included in the analyses. Several constructs considered in this study are conceptually related. We constructed the initial variable set using a structured selection process. First, potentially redundant or conceptually overlapping variables were identified as in the MOSAICH questionnaire, several items were used to operationalize one construct (e.g. smoking cigarettes, drinking alcohol, doing sport and eating vegetables or fruit to measure “health behaviour”). We only retained one representative variable (see line XX: “*We selected the item with the highest correlation with the target construct for inclusion in the analyses.”).* Afterwards, remaining multicollinearity was assessed using descriptive statistics and scatterplots.

-Given the reduction in sample size across analytical steps, It is necessary conducting sensitivity analyses to evaluate the impact of missing data, ideally considering imputation prior to model selection.

Thank you for this comment. You raised an important point, we used multiple imputation in the final analysis but used the available case population for the variable selection process. However, combining multiple imputation with penalized variable selection remains methodologically challenging, particularly regarding variable selection stability and pooling across imputed datasets. Given that simple imputation methods (like mean imputation) are not a good option, we think that our approach is reasonable. One could see the variable selection as a preliminary screening procedure, whereas effect estimation is based on the multiply imputed data.

-The definition and operationalization of “pet ownership” and animal contact should be more clearly justified, as the current approach aggregates heterogeneous exposures that may differ meaningfully in their relationship with the outcomes.

We totally agree with you that pet ownership is an aggregation of different exposures within one category. For this, we lay the foundation in the introduction section by saying that *“Different explanations have been proposed to explain the heterogeneity in findings, including weaknesses in research methodology and the complex nature of* *human–animal relationships, suggesting that associations between pet ownership and health may depend on multiple interacting factors related to the owner, the animal, and the human–animal relationship*” *(*line 146*)* and that “*Variability in findings may further reflect differences between animal species and patterns of interaction.”* (line 196).

In the methods section, we specify our definition and operationalization of pet ownership, see line 346: *Hart [115] outlined the diversity of the definitions of the term pet. Conservative definitions of pet include only dogs and cats. Other authors also include non-domesticated but tamed animals such as parrots. While some authors include only animals living in the same household as the owner, others also consider animals living outside the household, such as horses or chickens, to be pets.* *In our study, we defined pet as an animal living in the same household with a person. We also thus included non-domesticated animals such as birds or pet insects. As Hayama, Chang, Gumus, King, and Ernst [116] showed, pet ownership cannot be unequivocally answered with a single question. We therefore assessed pet ownership (defined as living with an animal in the household) and also regular contact with animals outside the household with two questions each.*

Moreover, we enlarged the description by adding the following statement in line 356: *We thus used a dimensional assessment of pet ownership and animal contact and further assessed the type of animal, average time spent with an animal per day, amount of contact with the closest animal as well as socio-emotional support from and attachment to animal.*

-Greater caution is also needed in the interpretation of the findings, particularly in light of the cross-sectional design and generally small effect sizes observed.

Thank you for raising this important point. We agree with you that interpretation of our findings need caution. We extended the discussion of the limitations as follows:

Line 1006: *Several limitations of this study should be considered. First, due to the cross-sectional design of this study, it was only possible to identify associations of pet ownership and regular animal contact with health and well-being measures. Causal conclusions could not and cannot be made. The observed associations may reflect causal effects, selection processes, or compensatory mechanisms, whereby individuals with specific psychological or social characteristics are more likely to acquire pets or seek animal contact. Longitudinal and causal modelling approaches are therefore needed to better disentangle these mechanisms and clarify whether pet ownership and animal contact influence health and well-being or whether pre-existing health characteristics influence the likelihood of acquiring pets. Moreover, caution should be taken in generalizing these results, as our analyses were exploratory, with several tested hypotheses and no correction in alpha error. Our results should be replicated and extended with other samples.*

Moreover, we again carefully checked for language regarding small effect sizes. We found it especially relevant that the result that pet owners smoke ‘0.19 cigarettes more per day’ is statistically significant but is a very small effect. As we used the difference as effect size, we changed the wording and added “slightly” throughout the manuscript:

Line 575: *…significantly but only slightly higher frequency of unhealthy behaviour…*

Line 737: *…reported a slightly higher frequency of unhealthy behaviour (operationalized as the number of smoked cigarettes per day)…*

Line 813: *… a slightly unhealthier behaviour…*

Line 817*: At the same time, the observed difference in smoking behaviour was minimal, amounting to only 0.19 additional cigarettes per day on average. The effect on unhealthy behaviour must be interpreted cautiously: If this is calculated over the period of a year, this makes a difference of 70 cigarettes, but such a small difference may not reflect a meaningful lifestyle disparity.*

Line 828: *… slightly more unhealthy behaviour…*

Line 1125*: While pet owners reported a slightly lower health status and slightly more unhealthy behaviour…*
