# Supplementary material for: Factors of pet ownership associated with human health: A representative cross-sectional survey of Switzerland
Source: PLoS One. 2026 Jul 29;21(7):e0352778. doi: 10.1371/journal.pone.0352778 (PMC13419196; doi:10.1371/journal.pone.0352778)
Supplement: Peer review history

# Peer review: Decision Letter 0

**DOI:** 10.1371/journal.pone.0352778.r001

**Authors:** Bibi Razieh Hosseini Farash

2 Jan 2026

PONE-D-25-39709

Factors of pet ownership associated with human health: a representative cross-sectional survey of Switzerland

PLOS One

Dear Dr. Hediger,

Thank you for submitting your manuscript to PLOS ONE. After careful consideration, we feel that it has merit but does not fully meet PLOS ONE’s publication criteria as it currently stands. Therefore, we invite you to submit a revised version of the manuscript that addresses the points raised during the review process.

Please submit your revised manuscript by Feb 16 2026 11:59PM. If you will need more time than this to complete your revisions, please reply to this message or contact the journal office at plosone@plos.org. When you're ready to submit your revision, log on to https://www.editorialmanager.com/pone/ and select the 'Submissions Needing Revision' folder to locate your manuscript file.

Please include the following items when submitting your revised manuscript:

- A letter that responds to each point raised by the academic editor and reviewer(s). You should upload this letter as a separate file labeled 'Response to Reviewers'.
- A marked-up copy of your manuscript that highlights changes made to the original version. You should upload this as a separate file labeled 'Revised Manuscript with Track Changes'.
- An unmarked version of your revised paper without tracked changes. You should upload this as a separate file labeled 'Manuscript'.

If you would like to make changes to your financial disclosure, please include your updated statement in your cover letter. Guidelines for resubmitting your figure files are available below the reviewer comments at the end of this letter.

If applicable, we recommend that you deposit your laboratory protocols in protocols.io to enhance the reproducibility of your results. Protocols.io assigns your protocol its own identifier (DOI) so that it can be cited independently in the future. For instructions see: https://journals.plos.org/plosone/s/submission-guidelines#loc-laboratory-protocols. Additionally, PLOS ONE offers an option for publishing peer-reviewed Lab Protocol articles, which describe protocols hosted on protocols.io. Read more information on sharing protocols at https://plos.org/protocols?utm_medium=editorial-email&utm_source=authorletters&utm_campaign=protocols.

We look forward to receiving your revised manuscript.

Kind regards,

Bibi Razieh Hosseini Farash

Academic Editor

PLOS One

**Journal Requirements:**

When submitting your revision, we need you to address these additional requirements.

1. Please ensure that your manuscript meets PLOS ONE's style requirements, including those for file naming. The PLOS ONE style templates can be found at

https://journals.plos.org/plosone/s/file?id=wjVg/PLOSOne_formatting_sample_main_body.pdf and

https://journals.plos.org/plosone/s/file?id=ba62/PLOSOne_formatting_sample_title_authors_affiliations.pdf

2. We noted in your submission details that a portion of your manuscript may have been presented or published elsewhere.

“No.

(some of the data we used are part of a publicly open national dataset and might have been used in other projects that we are not aware of).”

Please clarify whether this [conference proceeding or publication] was peer-reviewed and formally published. If this work was previously peer-reviewed and published, in the cover letter please provide the reason that this work does not constitute dual publication and should be included in the current manuscript.

3. When completing the data availability statement of the submission form, you indicated that you will make your data available on acceptance. We strongly recommend all authors decide on a data sharing plan before acceptance, as the process can be lengthy and hold up publication timelines. Please note that, though access restrictions are acceptable now, your entire data will need to be made freely accessible if your manuscript is accepted for publication. This policy applies to all data except where public deposition would breach compliance with the protocol approved by your research ethics board. If you are unable to adhere to our open data policy, please kindly revise your statement to explain your reasoning and we will seek the editor's input on an exemption. Please be assured that, once you have provided your new statement, the assessment of your exemption will not hold up the peer review process.

4. Please include captions for your Supporting Information files at the end of your manuscript, and update any in-text citations to match accordingly. Please see our Supporting Information guidelines for more information: http://journals.plos.org/plosone/s/supporting-information.

5. If the reviewer comments include a recommendation to cite specific previously published works, please review and evaluate these publications to determine whether they are relevant and should be cited. There is no requirement to cite these works unless the editor has indicated otherwise.

[Note: HTML markup is below. Please do not edit.]

Reviewers' comments:

Reviewer's Responses to Questions

**Comments to the Author**

1. Is the manuscript technically sound, and do the data support the conclusions?

The manuscript must describe a technically sound piece of scientific research with data that supports the conclusions. Experiments must have been conducted rigorously, with appropriate controls, replication, and sample sizes. The conclusions must be drawn appropriately based on the data presented.

Reviewer #1: Yes

Reviewer #2: Partly

**********

2. Has the statistical analysis been performed appropriately and rigorously?

Reviewer #1: Yes

Reviewer #2: Yes

**********

3. Have the authors made all data underlying the findings in their manuscript fully available?

The PLOS Data policy requires authors to make all data underlying the findings described in their manuscript fully available without restriction, with rare exception (please refer to the Data Availability Statement in the manuscript PDF file). The data should be provided as part of the manuscript or its supporting information, or deposited to a public repository. For example, in addition to summary statistics, the data points behind means, medians and variance measures should be available. If there are restrictions on publicly sharing data—e.g. participant privacy or use of data from a third party—those must be specified.

Reviewer #1: Yes

Reviewer #2: Yes

**********

4. Is the manuscript presented in an intelligible fashion and written in standard English?

PLOS ONE does not copyedit accepted manuscripts, so the language in submitted articles must be clear, correct, and unambiguous. Any typographical or grammatical errors should be corrected at revision, so please note any specific errors here.

Reviewer #1: Yes

Reviewer #2: Yes

**********

5. Review Comments to the Author

Please use the space provided to explain your answers to the questions above. You may also include additional comments for the author, including concerns about dual publication, research ethics, or publication ethics. (Please upload your review as an attachment if it exceeds 20,000 characters)

Reviewer #1: This study examines how pet ownership and regular animal contact status affect psychological well-being, life satisfaction, social support, loneliness, and health. My observations are given below:

(1) The study title is okay. The abstract needs some revisions. The abstract is well-structured and understandable, but it is too descriptive and doesn't concentrate on the theoretical justification or implications. The mechanical summary of statistical results does not emphasize significant levels or important patterns. The general conclusion provides little understanding of the underlying mechanisms or application.

(2) The introduction needs revisions. A thorough and well-structured review of the literature on the connection between pet ownership and human health and wellbeing is given in the introduction. It successfully demonstrates the importance of the subject and points out discrepancies in previous studies. But the main point is weakened by its excessive length and occasional repetition. Although stated, the justification for this study could be made clearer. Increased theoretical foundation and conceptual integration would improve coherence and make it clearer how this research advances our knowledge of human-animal relationships.

(3) The methods section needs modifications. The methods section improves representativeness and replicability by showcasing meticulous sampling and open data collection practices using the MOSAiCH survey. However, single-item measures for complex constructs (e.g., causal inference) are limited using cross-sectional design. A. loneliness, wellbeing) could diminish validity and dependability. It runs the risk of missing conceptual variance if only highly correlated items are chosen. While the statistical rigor demonstrated by LASSO regression and imputation is praiseworthy, interpretability is weakened by overfitting and a lack of theoretical support for variable inclusion. Methodological robustness may be enhanced by longitudinal or mixed-method approaches.

(4) The results require some modifications. Although there is a lot of descriptive and inferential information in the results section, the interpretation is not clear or succinct. Even though the statistical reporting is thorough, the results mostly show weak or non-significant associations, which raises concerns about their practical applicability. Clearer integration of descriptive and inferential findings and a targeted summary emphasizing important trends would improve the results. The rationale for controlling particular covariates should be supported, and the inclusion of many variables without a clear explanation suggests overcomplexity. The results are comprehensive overall, but they are not sufficiently synthesized to allow for meaningful interpretation.

(5) The discussion needs some revisions. It is thorough and well-structured, but it frequently repeats findings and is unduly descriptive rather than delving deeper into theoretical interpretation. Critical synthesis to explain discrepancies or contextual moderators, like socioeconomic factors or cultural attitudes toward pets, is lacking in this section, even though comparisons with previous research are properly cited. Although the causal ambiguity is recognized, the limitations are frequently brought up. More integrative implications for future research could be made, emphasizing theoretical or applied contributions as opposed to just methodological improvements. Clarity is generally good, but there is little analytical depth.

(6) Please modify and check reference styles aper the journal guidelines.

Reviewer #2:

1. While the introduction details inconsistent findings from past research on the pet effect (some beneficial, some not, some harmful), it lacks an integrative psychological framework to explain these discrepancies. Attributing the results solely to methodological weaknesses or demographic differences is insufficient.

2. Your results suggest that higher socio-emotional support from animals is associated with higher levels of loneliness. Psychologically, does this imply a compensatory mechanism rather than a causal one? In other words, do lonely individuals seek stronger emotional bonds with pets to compensate for a lack of human social support?

3. Research has found that pet owners report slightly lower levels of health and more frequent unhealthy behaviors such as smoking. Can you elaborate on the selection effect in the discussion? Specifically, from a health psychology perspective, are individuals with pre-existing health problems or higher levels of smoking-related stress more likely to own pets for comfort or companionship?

4. I noticed that constructs such as loneliness and mental health are measured using single-item measures. From a psychometric perspective, single-item measures generally have lower reliability and validity than existing scales. 5. Does the limited variance captured by these single items affect the results? Please elaborate on how this measurement limitation affects the sensitivity of your results.

5. The result that pet owners "smoke 0.19 more cigarettes per day" is statistically significant but seems to lack practical or clinical significance. Calling this an "unhealthy behavior" finding seems somewhat exaggerated. The authors should provide an effect size (e.g., Cohen's d) or more cautiously interpret this magnitude, acknowledging that such a small difference may not reflect substantial lifestyle differences.

6. Combining "horses" with "farm animals" and "rodents" with "birds" ensures that the sample size may dilute species-specific effects. From a psychological perspective, interactions with horses (typically involving movement and high engagement) differ significantly from interactions with farm livestock. This limitation may explain invalid results regarding species and should be acknowledged as a factor limiting the ability of studies to detect species-specific benefits.

7. The finding that regular contact with animals in ecospheres/aquariums is associated with lower mental health is interesting. What could be the reason from an evolutionary psychology or environmental psychology perspective? Does this reflect the solitary nature of such hobbies, or do the psychological motivations for keeping non-interactive pets differ from those for social mammals like dogs? Expanding the psychological functions across different species would add depth to your discussion of species-specific effects.

8. In your discussion, you suggested that sociodemographic differences might explain the absence of the pet effect. However, your regression model, controlling for these variables (sex, age, settlement type, etc.), still found a negative correlation with health status. The discussion needs to address why this negative association persists after adjustment. This suggests the presence of other factors or unmeasured confounding factors beyond just demographics.

**********

6. PLOS authors have the option to publish the peer review history of their article (what does this mean?). If published, this will include your full peer review and any attached files.

If you choose “no”, your identity will remain anonymous but your review may still be made public.

**Do you want your identity to be public for this peer review?**  For information about this choice, including consent withdrawal, please see our Privacy Policy.

Reviewer #1: **Yes:** Gyanesh Kumar Tiwari

Reviewer #2: **Yes:** Huan Qi

**********

[NOTE: If reviewer comments were submitted as an attachment file, they will be attached to this email and accessible via the submission site. Please log into your account, locate the manuscript record, and check for the action link "View Attachments". If this link does not appear, there are no attachment files.]

To ensure your figures meet our technical requirements, please review our figure guidelines: https://journals.plos.org/plosone/s/figures

You may also use PLOS’s free figure tool, NAAS, to help you prepare publication quality figures: https://journals.plos.org/plosone/s/figures#loc-tools-for-figure-preparation.

NAAS will assess whether your figures meet our technical requirements by comparing each figure against our figure specifications.

Attachment Submitted filename: comment.docx

# Author comment: Author response to Decision Letter 1

**DOI:** 10.1371/journal.pone.0352778.r002

18 Feb 2026

1. While the introduction section meticulously enumerates the inconsistent findings of past research on the pet effect (some beneficial, some non-beneficial or harmful), it lacks an integrative psychological theoretical framework to explain these discrepancies. Simply attributing the results to methodological weaknesses or demographic differences is insufficient.

Thank you for this important comment. We agree that a psychological theoretical framework is essential to explain the discrepancies observed in our findings. Accordingly, we discuss several relevant frameworks such as selection processes (e.g., healthier or less healthy individuals being more likely to adopt a pet), attachment processes as potential moderators (starting at line 138), and other psychological factors such as mindfulness experienced in the presence of pets (line 156). Based on these considerations, we argue that research should account not only for sociodemographic factors but also for social support, the amount of contact with the animal, and attachment to the animal.

It is possible that these psychological theoretical frameworks were not sufficiently clear to readers. To address this, we have extended the Introduction by adding the following sentences:

Line 128: This raises the question of whether pet ownership leads to changes in health, or whether individuals with better or poorer physical or mental health are more likely to acquire pets. In the latter case, observed associations would not reflect causal effects but rather underlying selection processes.

Line 141: The heterogeneity in results can also be explained by further psychological theoretical frameworks, such as attachment processes and social support theory. Loneliness or living alone can have a mediating role, driving another selection process (98). It has been hypothesized that individuals who experience loneliness, anxiety, or depressive symptoms may be more likely to adopt a pet as a form of self-medication, indicating a compensatory mechanism (99) whereby, e.g., lonely individuals seek stronger emotional bonds with pets to compensate a lack of human social support.

2. Your results indicate that higher socio-emotional support from animals is associated with higher levels of loneliness. From a psychological perspective, could this suggest a compensatory mechanism rather than a causal one? In other words, do lonely individuals seek out stronger emotional bonds with pets to compensate for a lack of human social support?

Thank you for this helpful comment. Indeed, this is a mechanism that is often hypothesized. We have already inserted this according to your previous comment (see above):

Line 143: It has been hypothesized that individuals who experience loneliness, anxiety, or depressive symptoms may be more likely to adopt a pet as a form of self-medication, indicating a compensatory mechanism (99) whereby, e.g., lonely individuals seek stronger emotional bonds with pets to compensate a lack of human social support.

3. The study finds that pet owners reported slightly lower health status and higher frequency of unhealthy behaviors like smoking. Could you elaborate more on the selection effect in your discussion? Specifically, from a health psychology standpoint, is it plausible that individuals with pre-existing health issues or higher stress levels (correlated with smoking) are more likely to acquire pets for comfort or companionship?

Thank you for your helpful comment; you are absolutely right. We have now included this point in the Discussion section, following the corresponding addition to the Introduction:

Line 882: We hypothesize that this negative association could reflect a selection effect. Individuals with pre-existing health issues or higher stress levels, which are correlated with smoking (124), might be more likely to adopt a pet for comfort or companionship as a compensatory mechanism.

4. I note that psychological constructs such as loneliness and psychological well-being were measured using single items. From a psychometric perspective, single-item measures often have lower reliability and validity compared to established scales. Could the limited variance captured by these single items influence the result? Please add a discussion on how this measurement limitation might affect the sensitivity of your results.

Thank you for this important comment. We agree with you that single-item measures can have weaknesses. However, they can also have strengths as outlined by the editors of the World Happiness Report 2025 who explained in their article why single-item measures of wellbeing are best (Helliwell, J.F., Layard, R., Sachs, J.D. et al. (2026). Why single-item measures of wellbeing are best. Nature Human Behavior. https://doi.org/10.1038/s41562-026-02401-y). But we acknowledge the debate and extended the limitations section as follows:

Line 1081: We acknowledge the ongoing debate regarding the reliability and validity of single-item measures (132) for assessing complex psychological constructs such as loneliness and well-being. While some studies suggest that single-item measures have comparable validity to multiple-item scales (133,134) and may be suitable for assessing well-being (135), the limitations of single-item measures must also be considered. It is therefore possible that our measurement approach was not sufficiently sensitive to detect important differences.

5. The result that pet owners smoke ‘0.19 cigarettes more per day’ is statistically significant but appears to lack practical or clinical significance. Emphasizing this as a finding of ‘unhealthy behavior’ seems overstated. The authors should provide effect sizes (e.g., Cohen's d) or interpret this magnitude with more caution, acknowledging that such a small difference may not reflect a meaningful lifestyle disparity.

Thank you for raising this important point. We agree with you that this is a very small effect. As we used the difference as effect size, we changed the wording and added “slightly” throughout the manuscript. Moreover, we extended the limitations:

Line 725: …significantly but only slightly higher frequency of unhealthy behaviour…

Line 862: …reported a slightly higher frequency of unhealthy behaviour (operationalized as the number of smoked cigarettes per day)…

Line 878: …, apart from a slightly higher frequency of smoking cigarettes in pet owners compared to non-pet owners.

Line 879: The effect on unhealthy behaviour must be interpreted cautiously as the number of smoked cigarettes per day only differed by 0.19 cigarettes more per day. If this is calculated over the period of a year, this makes a difference of 70 cigarettes, but such a small difference may not reflect a meaningful lifestyle disparity.

Line 925: … a slightly unhealthier behaviour…

Line 928: … a slightly unhealthier behaviour…

6. The aggregation of ‘Equines’ with ‘Farm animals’ and ‘Rodents’ with ‘Birds’ to ensure sample size may dilute species-specific effects. Psychologically, the interaction with a horse (often involving sport and high engagement) is vastly different from interacting with farm livestock. This limitation might explain the null results regarding species and should be acknowledged as a constraint on the study's ability to detect species-specific benefits.

Thank you for this important comment. We agree that it would have been interesting to analyze the animal species separately. However, the number of observations for some species was too small, resulting in highly uneven category distributions, which would have posed challenges for the statistical models. For example, we only had 26 animals in the category “farm animals and equines” together. We therefore aimed to define broader categories of animal species that share at least some common characteristics regarding interaction. As we controlled for the amount of time spent and attachment to the pet, some of the effects are diminished, but still, this aggregation may have led to the null results. This limitation has been acknowledged in the manuscript as follows:

Line 1092: To achieve more balanced category distributions, we aggregated certain animal species (e.g., rodents and birds, as well as farm animals and equines) into broader categories. However, interactions with horses, for example, can differ substantially from interactions with other farm animals, which may have contributed to the null findings for species effects, despite controlling for time spent with the animal and attachment to the animal.

7. The finding that regular contact with animals kept in terrariums/aquariums was associated with lower psychological well-being is intriguing. From an evolutionary or environmental psychology perspective, what might explain this? Does this reflect the solitary nature of such hobbies, or perhaps a different psychological motivation for keeping non-interactive pets compared to social mammals like dogs? Expanding on the psychological function of different species would add depth to your discussion on species-specific effects.

Thank you for this comment. We also find this result interesting and happily extended our discussion:

Line 961: The finding regarding animals kept in a terrarium or aquarium raises important questions about the mechanisms underlying species-specific human-animal interactions. From an attachment and social support perspective, the benefits of animal contact are often attributed to social interaction, emotional reciprocity, and perceived responsiveness (125), which are more characteristic of socially interactive mammals than of animals typically kept in terrariums or aquariums. Consequently, contact with less interactive species may not engage the same attachment-related or social buffering mechanisms associated with psychological well-being. This aligns with developmental findings showing that children’s empathy toward animals tends to decrease with increasing phylogenetic distance (126), suggesting that social closeness and perceived similarity may shape emotional responses to other species. An alternative explanation for the observed association may reflect selection effects: individuals who prefer contact with non-interactive animals may differ systematically in personality traits, social needs, or lifestyle factors that themselves might relate to lower well-being. Whereas keeping dogs or cats may serve compensatory social or emotional functions, keeping terrarium or aquarium animals may primarily fulfil other motives (e.g., fascination with exotic species, aesthetic appreciation, or a preference for low-maintenance companionship) (127), which may not offer the same socioemotional benefits. Nevertheless, a survey on reptile owners found that an emotional attachment toward reptiles was within the range of those observed in traditional pets (128). Together, these findings underscore the importance of considering species-specific interaction styles and individual differences when evaluating well-being and pet ownership.

8. In the Discussion, you suggest that socio-demographic differences might explain the lack of a pet effect. However, your regression models controlled for these variables (gender, age, settlement type, etc.) and still found a negative association with health status. The discussion needs to address why the negative association persists after adjustment. This suggests if there existed some other factors or unmeasured confounders, rather than just demographics.

You are correct. While our models controlled for key socio-demographic factors, the negative association between pet ownership and health status persisted. This is addressed in the discussion from line 925 on. We have extended the arguments to make clear that beyond socio-demographic factors, compensatory mechanisms and the selection effect might be relevant in explaining our results.

Line 928: Another possibility is that unhealthier people are more likely to acquire pets as a compensatory mechanism and that the results can be explained by a selection effect, even though the literature largely argues that healthier people are more likely to acquire pets.

Attachment Submitted filename: Point-by-point-response_Revision.docx

# Peer review: Decision Letter 1

**DOI:** 10.1371/journal.pone.0352778.r003

**Authors:** Vinícius Silva Belo

15 Apr 2026

PONE-D-25-39709R1

Factors of pet ownership associated with human health: a representative cross-sectional survey of Switzerland

PLOS One

Dear Dr. Hediger,

Thank you for submitting your manuscript to PLOS ONE. After careful consideration, we feel that it has merit but does not fully meet PLOS ONE’s publication criteria as it currently stands. Therefore, we invite you to submit a revised version of the manuscript that addresses the points raised during the review process.

**As the newly assigned Academic Editor, I have carefully evaluated the revised version of your manuscript. While the revision has addressed several points raised in the previous round important issues remain unresolved.**

**First, I was unable to identify a clear, point-by-point response to the comments raised by Reviewer #1 in the previous round. In addition, several of the concerns raised by this reviewer do not appear to have been adequately incorporated into the revised manuscript. For transparency, I am including the full set of comments from Reviewer #1 below. Please provide a structured, point-by-point response and revise the manuscript accordingly.**

***This study examines how pet ownership and regular animal contact status affect psychological well-being, life satisfaction, social support, loneliness, and health. My observations are given below:***

***(1) The study title is okay. The abstract needs some revisions. The abstract is well-structured and understandable, but it is too descriptive and doesn't concentrate on the theoretical justification or implications. The mechanical summary of statistical results does not emphasize significant levels or important patterns. The general conclusion provides little understanding of the underlying mechanisms or application.***

***(2) The introduction needs revisions. A thorough and well-structured review of the literature on the connection between pet ownership and human health and wellbeing is given in the introduction. It successfully demonstrates the importance of the subject and points out discrepancies in previous studies. But the main point is weakened by its excessive length and occasional repetition. Although stated, the justification for this study could be made clearer. Increased theoretical foundation and conceptual integration would improve coherence and make it clearer how this research advances our knowledge of human-animal relationships.***

***(3) The methods section needs modifications. The methods section improves representativeness and replicability by showcasing meticulous sampling and open data collection practices using the MOSAiCH survey. However, single-item measures for complex constructs (e.g., causal inference) are limited using cross-sectional design. A. loneliness, wellbeing) could diminish validity and dependability. It runs the risk of missing conceptual variance if only highly correlated items are chosen. While the statistical rigor demonstrated by LASSO regression and imputation is praiseworthy, interpretability is weakened by overfitting and a lack of theoretical support for variable inclusion. Methodological robustness may be enhanced by longitudinal or mixed-method approaches.***

***(4) The results require some modifications. Although there is a lot of descriptive and inferential information in the results section, the interpretation is not clear or succinct. Even though the statistical reporting is thorough, the results mostly show weak or non-significant associations, which raises concerns about their practical applicability. Clearer integration of descriptive and inferential findings and a targeted summary emphasizing important trends would improve the results. The rationale for controlling particular covariates should be supported, and the inclusion of many variables without a clear explanation suggests overcomplexity. The results are comprehensive overall, but they are not sufficiently synthesized to allow for meaningful interpretation.***

***(5) The discussion needs some revisions. It is thorough and well-structured, but it frequently repeats findings and is unduly descriptive rather than delving deeper into theoretical interpretation. Critical synthesis to explain discrepancies or contextual moderators, like socioeconomic factors or cultural attitudes toward pets, is lacking in this section, even though comparisons with previous research are properly cited. Although the causal ambiguity is recognized, the limitations are frequently brought up. More integrative implications for future research could be made, emphasizing theoretical or applied contributions as opposed to just methodological improvements. Clarity is generally good, but there is little analytical depth.***

***(6) Please modify and check reference styles as per the journal guidelines.***

***In addition to the points above, I would like to highlight several aspects that require attention from an editorial perspective. The limitations inherent to the cross-sectional design should be more clearly emphasized, particularly regarding the inability to establish causality and the possibility of reverse causation and selection effects. The use of single-item measures for complex constructs such as loneliness and psychological well-being should also be more critically discussed, considering potential limitations in validity and sensitivity.***

**Furthermore:**

**-The analytical strategy would benefit from additional clarification, including a more explicit justification for the inclusion of covariates and an assessment of potential collinearity among predictors.**

**-Given the reduction in sample size across analytical steps, It is necessary conducting sensitivity analyses to evaluate the impact of missing data, ideally considering imputation prior to model selection.**

**-The definition and operationalization of “pet ownership” and animal contact should be more clearly justified, as the current approach aggregates heterogeneous exposures that may differ meaningfully in their relationship with the outcomes.**

**-Greater caution is also needed in the interpretation of the findings, particularly in light of the cross-sectional design and generally small effect sizes observed.**

Please submit your revised manuscript by May 30 2026 11:59PM. If you will need more time than this to complete your revisions, please reply to this message or contact the journal office at plosone@plos.org. When you're ready to submit your revision, log on to https://www.editorialmanager.com/pone/ and select the 'Submissions Needing Revision' folder to locate your manuscript file.

Please include the following items when submitting your revised manuscript:

- A letter that responds to each point raised by the academic editor and reviewer(s). You should upload this letter as a separate file labeled 'Response to Reviewers'.
- A marked-up copy of your manuscript that highlights changes made to the original version. You should upload this as a separate file labeled 'Revised Manuscript with Track Changes'.
- An unmarked version of your revised paper without tracked changes. You should upload this as a separate file labeled 'Manuscript'.

If you would like to make changes to your financial disclosure, please include your updated statement in your cover letter. Guidelines for resubmitting your figure files are available below the reviewer comments at the end of this letter.

If applicable, we recommend that you deposit your laboratory protocols in protocols.io to enhance the reproducibility of your results. Protocols.io assigns your protocol its own identifier (DOI) so that it can be cited independently in the future. For instructions see: https://journals.plos.org/plosone/s/submission-guidelines#loc-laboratory-protocols. Additionally, PLOS ONE offers an option for publishing peer-reviewed Lab Protocol articles, which describe protocols hosted on protocols.io. Read more information on sharing protocols at https://plos.org/protocols?utm_medium=editorial-email&utm_source=authorletters&utm_campaign=protocols.

As the corresponding author, your ORCID iD is verified in the submission system and will appear in the published article. PLOS supports the use of ORCID, and we encourage all coauthors to register for an ORCID iD and use it as well. Please encourage your coauthors to verify their ORCID iD within the submission system before final acceptance, as unverified ORCID iDs will not appear in the published article. *Only*  the individual author can complete the verification step; PLOS staff *cannot*  verify ORCID iDs on behalf of authors.

We look forward to receiving your revised manuscript.

Kind regards,

Vinícius Silva Belo

Academic Editor

PLOS One

Journal Requirements:

If the reviewer comments include a recommendation to cite specific previously published works, please review and evaluate these publications to determine whether they are relevant and should be cited. There is no requirement to cite these works unless the editor has indicated otherwise.

[Note: HTML markup is below. Please do not edit.]

Reviewers' comments:

Reviewer's Responses to Questions

**Comments to the Author**

1. If the authors have adequately addressed your comments raised in a previous round of review and you feel that this manuscript is now acceptable for publication, you may indicate that here to bypass the “Comments to the Author” section, enter your conflict of interest statement in the “Confidential to Editor” section, and submit your "Accept" recommendation.

Reviewer #1: All comments have been addressed

**********

2. Is the manuscript technically sound, and do the data support the conclusions?

The manuscript must describe a technically sound piece of scientific research with data that supports the conclusions. Experiments must have been conducted rigorously, with appropriate controls, replication, and sample sizes. The conclusions must be drawn appropriately based on the data presented.

Reviewer #1: Yes

**********

3. Has the statistical analysis been performed appropriately and rigorously?

Reviewer #1: Yes

**********

4. Have the authors made all data underlying the findings in their manuscript fully available?

The PLOS Data policy requires authors to make all data underlying the findings described in their manuscript fully available without restriction, with rare exception (please refer to the Data Availability Statement in the manuscript PDF file). The data should be provided as part of the manuscript or its supporting information, or deposited to a public repository. For example, in addition to summary statistics, the data points behind means, medians and variance measures should be available. If there are restrictions on publicly sharing data—e.g. participant privacy or use of data from a third party—those must be specified.

Reviewer #1: Yes

**********

5. Is the manuscript presented in an intelligible fashion and written in standard English?

PLOS ONE does not copyedit accepted manuscripts, so the language in submitted articles must be clear, correct, and unambiguous. Any typographical or grammatical errors should be corrected at revision, so please note any specific errors here.

Reviewer #1: Yes

**********

6. Review Comments to the Author

Please use the space provided to explain your answers to the questions above. You may also include additional comments for the author, including concerns about dual publication, research ethics, or publication ethics. (Please upload your review as an attachment if it exceeds 20,000 characters)

Reviewer #1: I took another close look at the manuscript. I am pleased with the authors' efforts to make revisions to the work. I believe the writers have answered every query posed in the initial review. The manuscript has been revised to be more comprehensive and readable. Its technical fit has since improved. I think the manuscript, in its current state, has the potential to advance knowledge.

**********

7. PLOS authors have the option to publish the peer review history of their article (what does this mean?). If published, this will include your full peer review and any attached files.

If you choose “no”, your identity will remain anonymous but your review may still be made public.

**Do you want your identity to be public for this peer review?**  For information about this choice, including consent withdrawal, please see our Privacy Policy.

Reviewer #1: **Yes:** Gyanesh Kumar Tiwari

**********

[NOTE: If reviewer comments were submitted as an attachment file, they will be attached to this email and accessible via the submission site. Please log into your account, locate the manuscript record, and check for the action link "View Attachments". If this link does not appear, there are no attachment files.]

To ensure your figures meet our technical requirements, please review our figure guidelines: https://journals.plos.org/plosone/s/figures

You may also use PLOS’s free figure tool, NAAS, to help you prepare publication quality figures: https://journals.plos.org/plosone/s/figures#loc-tools-for-figure-preparation.

NAAS will assess whether your figures meet our technical requirements by comparing each figure against our figure specifications.

# Author comment: Author response to Decision Letter 2

**DOI:** 10.1371/journal.pone.0352778.r004

30 May 2026

(1) The study title is okay. The abstract needs some revisions. The abstract is well-structured and understandable, but it is too descriptive and doesn't concentrate on the theoretical justification or implications. The mechanical summary of statistical results does not emphasize significant levels or important patterns. The general conclusion provides little understanding of the underlying mechanisms or application.

We thank the reviewer for this thoughtful comment. We agree with you that theoretical justification is important. We thus added the following sentence to emphasize the theoretical justification, see line 21: “It is suggested that factors related to the owner, the animal and the human–animal relationship might explain some of the previous heterogeneity in findings.”

As there is a word limit for the abstract and the effect sizes are small, we decided to use the effect wording and not to present the significance levels within the results to avoid overinterpretation. However, if the editor would like us to add the estimates and CI’s together with the p-values, we are happy to include these also in the abstract.

We were cautious to not overinterpreting our findings and did not want to imply anything into the data that cannot be justified as assuming underlying mechanisms would be based on interpretation. However, we acknowledge the reviewer’s request and concern and added a the following text, see line 45: “We found little evidence for a general association between pet ownership or regular animal contact and human health and well-being. However, relationship-related factors, including socio-emotional support from the animal and the amount of contact with the animal, were sometimes associated with well-being and the quality of the human–animal relationship depends on different factors such as the amount of spent time or animal species. These findings suggest that associations between pet ownership and human health and well-being depend on characteristics of the owner, the animal and the human–animal relationship itself.”

(2) The introduction needs revisions. A thorough and well-structured review of the literature on the connection between pet ownership and human health and wellbeing is given in the introduction. It successfully demonstrates the importance of the subject and points out discrepancies in previous studies. But the main point is weakened by its excessive length and occasional repetition. Although stated, the justification for this study could be made clearer. Increased theoretical foundation and conceptual integration would improve coherence and make it clearer how this research advances our knowledge of human-animal relationships.

We thank the reviewer for this thoughtful and constructive comment. We agree that the introduction benefits from a clearer conceptual structure and stronger theoretical integration.

In response, we substantially revised and reorganized the introduction to improve coherence, reduce redundancy, and clarify the rationale and contribution of the study. First, we condensed several sections of the literature review, particularly the overview of positive effects of pet ownership, by synthesizing findings more compactly and reducing repetitive examples (see page 4-6). Second, we restructured the discussion of heterogeneity in previous findings into conceptually distinct sections addressing: (1) socio-demographic factors and selection processes, (2) attachment and social support theory, (3) animal species and interaction intensity, and (4) psychological and neurobiological mechanisms underlying human–animal relationships (see page 7-11). This restructuring was intended to strengthen the theoretical framework and improve the logical flow of the introduction.

In addition, we revised the study rationale to more explicitly emphasize that associations between pet ownership and health may depend not only on ownership status itself, but also on interacting owner-, animal-, and relationship-related factors, including socio-demographic characteristics, attachment to the animal, perceived socio-emotional support, amount of animal contact, and animal species. We also clarified that the present study advances previous research by examining these dimensions simultaneously within a nationally representative sample (see line 253).

Together, these revisions were intended to improve conceptual integration, reduce repetition, and more clearly position the study’s contribution to understanding the complexity of human–animal relationships and their associations with health and well-being.

(3) The methods section needs modifications. The methods section improves representativeness and replicability by showcasing meticulous sampling and open data collection practices using the MOSAiCH survey. However, single-item measures for complex constructs (e.g., causal inference) are limited using cross-sectional design. A. loneliness, wellbeing) could diminish validity and dependability. It runs the risk of missing conceptual variance if only highly correlated items are chosen. While the statistical rigor demonstrated by LASSO regression and imputation is praiseworthy, interpretability is weakened by overfitting and a lack of theoretical support for variable inclusion. Methodological robustness may be enhanced by longitudinal or mixed-method approaches.

Thank you for these important comments. The other reviewer has also pointed out the use of single-item measures. We agree with you that single-item measures can have weaknesses and can lead to potential missing conceptual variance. Of course, each dimension reduction approach if PCA or simply selecting one representative variable will reduce the variance of a high dimensional construct. We fear that there is not much we can do given the amount of potentially interesting variables. However, they can also have strengths as outlined by e.g. the editors of the World Happiness Report 2025 who explained in their article why single-item measures of wellbeing are best (Helliwell, J.F., Layard, R., Sachs, J.D. et al. (2026). Why single-item measures of wellbeing are best. Nature Human Behavior. https://doi.org/10.1038/s41562-026-02401-y). But we take the reviewers concern serious and acknowledge the debate and thus extended the limitations section as follows:

Line 1023: We acknowledge the ongoing debate regarding the reliability and validity of single-item measures (143) for assessing complex psychological constructs such as loneliness and well-being. While some studies suggest that single-item measures have comparable validity to multiple-item scales (144,145) and may be suitable for assessing well-being (146, the limitations of single-item measures must also be considered. It is therefore possible that our measurement approach was not sufficiently sensitive to detect important differences.

With respect to overfitting, we are a bit less worried than the reviewer. Of course, LASSO does not prevent overfitting similarly well than building and validating statistical models with different datasets, but LASSO combined with in-built cross-validation has in many simulation studies shown to substantially reduce overfitting. Anyway, we added the following sentence to the limitation section, see line 1029: The lasso procedure reduces overfitting by avoiding overly complex models. However, given the number of variables there is no guarantee that overfitting will be eliminated and – as always with variable selection – coefficients should be interpreted with care.

And to address the comment regarding enhancing methodological robustness by longitudinal or mixed-method approaches, we extended the suggestions for future research as follows (see line 1081): Future research should therefore investigate how humans interact with animals, what functions these relationships fulfil, and under which conditions they may contribute to health and well-being. Longitudinal studies are needed to clarify whether the associations found here represent causal effects or selection processes. Moreover, mixed-methods approaches might enhance methodological robustness.

(4) The results require some modifications. Although there is a lot of descriptive and inferential information in the results section, the interpretation is not clear or succinct. Even though the statistical reporting is thorough, the results mostly show weak or non-significant associations, which raises concerns about their practical applicability. Clearer integration of descriptive and inferential findings and a targeted summary emphasizing important trends would improve the results. The rationale for controlling particular covariates should be supported, and the inclusion of many variables without a clear explanation suggests overcomplexity. The results are comprehensive overall, but they are not sufficiently synthesized to allow for meaningful interpretation.

Thank you for your important comments. We address the two separate points as follows:

A) Integration of descriptive and inferential findings, a targeted summary with important trends and synthesizing. The reviewer states that the interpretation of the results is not clear or succinct. We acknowledge this point but decided not to add interpretations in the results section but rather interpret the findings in the discussion. However, we agree with the reviewer that the results section profited from clearer integration and synthetization. We added the following passages:

Line 545: “The descriptive statistics shows that pet owners or people with regular animal contact were in general four years younger and were more likely to be female compared to non-pet owners and people without regular animal contact. Their marital status was comparable, while pet owners or people with regular animal contact had completed vocational training slightly more often but possessed a degree from a university or a university of applied sciences less often. Pet owners or people with regular animal contact were also more likely to be employed or to be in education and were less likely to be retired compared to non-pet owners and people without regular animal contact. The presence of a partner and household income were comparable, while pet owners or people with regular animal contact were less likely to live in a household with one or two people and more likely to live in a household with three or more people. Moreover, pet owners or people with regular animal contact were less likely to live in a city, suburb, or town and more likely lived in a village or the countryside than non-pet owners and people without regular animal contact.”

Line 606: “Taken together, these findings show that pet owners reported a significantly lower self-reported health status and a slightly higher frequency of unhealthy behaviour compared to people without pets. People with and without regular animal contact did not differ in any of the health or well-being outcomes.”

Line 665: Taken together, these findings show that health status or healthcare use was not associated with attachment to animals, socio-emotional support from animal, amount of animal contact or animal species. However, a higher amount of animal contact was significantly associated with slightly more unhealthy behaviour. Higher life satisfaction was significantly associated with higher amount of regular contact with other species but not with other factors. Higher socio-emotional support from the animal was tendentially associated with a slightly higher loneliness and with lower psychological well-being. Compared to other outcomes, psychological wellbeing was associated with the most factors. Higher psychological well-being was tendentially associated with a higher amount of animal contact. Lower psychological well-being was tendentially associated with higher socio-emotional support from the animal and significantly associated with more regular contact with an animal kept in a terrarium or aquarium.”

Line 718: “Taken together, these findings show that the attachment to the animal and the socio-emotional support from the animal are associated with several characteristics of the owner such as gender or whether they were married, of the animal species and of the human-animal relationship such as the amount of animal contact or if the animal lives in the household or not.”

B) Rationale for the inclusion of predictors and covariates. A large part of the introduction functions as foundation or rationale for the inclusion of the variables and covariates that we use in our analyses. However, we agree that this can be made more explicit and added a rationale for the predictors in the methods section where we describe each theoretical model.

Line 451: These predictors were chosen based on literature showing that human health highly depends on perceived loneliness, human social support and psychological well-being (94,123).

Line 461: The predictor was chosen based on literature showing that loneliness is often closely linked to human social support (128).

Line 466: Predictors were chosen because they can all be closely linked to each other, as recent studies show (129,130).

Line 473: The choice of the predictors was based on literature (92,131,132).

Line 482: The choice of predictors had the same basis as mentioned above.

The basis for the rationale for inclusion of the covariates is given in the introduction. First, we introduce what socio-demographic variables are important according to the literature when it comes to physical and mental health and pet-ownership. See from line 158. Then, we explain why the intensity of the relationship and the strength of the attachment to the animal are important, as they may mediate the association between pet ownership and well-being (see line 183). Third, we introduce that animal species and patterns of interaction with the animal is an important aspect that may mediate the association between pet ownership and well-being (see from line 196).

However, we understand that it is helpful for readers to justify the inclusion of the specific covariates more clearly. To address this, we extended the following sentence:

Line 264: To address these aims, we analysed …. and controlled for different socio-demographic factors that have been identified as important in previous literature.

We also added the rationale for the use of age, gender, relationship status and social network, socioeconomic status, living area, educational level, employment status as socio-demographic variables in the methods section:

Line 330: These socio-demographic variables were chosen as previous literature has identified those as important influencing factors for human physical and mental health (1,2,4,7,79,92,93).

Line 456: The socio-demographic variables were chosen based on previous literature identifying them as important factors for human health (1,2,4,7,79,92,93).

(5) The discussion needs some revisions. It is thorough and well-structured, but it frequently repeats findings and is unduly descriptive rather than delving deeper into theoretical interpretation. Critical synthesis to explain discrepancies or contextual moderators, like socioeconomic factors or cultural attitudes toward pets, is lacking in this section, even though comparisons with previous research are properly cited. Although the causal ambiguity is recognized, the limitations are frequently brought up. More integrative implications for future research could be made, emphasizing theoretical or applied contributions as opposed to just methodological improvements. Clarity is generally good, but there is little analytical depth.

We thank the reviewer for this helpful comment. We substantially revised the discussion section to improve conceptual integration, analytical depth, and coherence. Specifically, we reduced redundancies and repetitive reporting of findings by combining several result sections and streamlining descriptive passages. We further restructured the discussion according to the conceptual topics introduced in the introduction, including three new sub-headings: The absence of the “pet effect” (from line 745), Ther role of the characteristics of the human-animal relationship (from line 843), and Conceptualizing underlying mechanisms (from line 96

Attachment Submitted filename: Point-by-point-response_Reviewer1_final.docx

# Peer review: Decision Letter 2

**DOI:** 10.1371/journal.pone.0352778.r005

**Authors:** Vinícius Silva Belo

16 Jun 2026

Factors of pet ownership associated with human health: a representative cross-sectional survey of Switzerland

PONE-D-25-39709R2

Dear Dr. Hediger,

We’re pleased to inform you that your manuscript has been judged scientifically suitable for publication and will be formally accepted for publication once it meets all outstanding technical requirements.

Within one week, you’ll receive an e-mail detailing the required amendments. When these have been addressed, you’ll receive a formal acceptance letter and your manuscript will be scheduled for publication.

An invoice will be generated when your article is formally accepted. Please note, if your institution has a publishing partnership with PLOS and your article meets the relevant criteria, all or part of your publication costs will be covered. Please make sure your user information is up-to-date by logging into Editorial Manager at Editorial Manager® and clicking the ‘Update My Information' link at the top of the page. For questions related to billing, please contact billing support.

If your institution or institutions have a press office, please notify them about your upcoming paper to help maximize its impact. If they’ll be preparing press materials, please inform our press team as soon as possible -- no later than 48 hours after receiving the formal acceptance. Your manuscript will remain under strict press embargo until 2 pm Eastern Time on the date of publication. For more information, please contact onepress@plos.org.

Kind regards,

Vinícius Silva Belo

Academic Editor

PLOS One

Additional Editor Comments (optional):

Reviewers' comments:

Reviewer's Responses to Questions

**Comments to the Author**

1. If the authors have adequately addressed your comments raised in a previous round of review and you feel that this manuscript is now acceptable for publication, you may indicate that here to bypass the “Comments to the Author” section, enter your conflict of interest statement in the “Confidential to Editor” section, and submit your "Accept" recommendation.

Reviewer #1: All comments have been addressed

**********

2. Is the manuscript technically sound, and do the data support the conclusions?

The manuscript must describe a technically sound piece of scientific research with data that supports the conclusions. Experiments must have been conducted rigorously, with appropriate controls, replication, and sample sizes. The conclusions must be drawn appropriately based on the data presented.

Reviewer #1: Yes

**********

3. Has the statistical analysis been performed appropriately and rigorously?

Reviewer #1: Yes

**********

4. Have the authors made all data underlying the findings in their manuscript fully available?

The PLOS Data policy requires authors to make all data underlying the findings described in their manuscript fully available without restriction, with rare exception (please refer to the Data Availability Statement in the manuscript PDF file). The data should be provided as part of the manuscript or its supporting information, or deposited to a public repository. For example, in addition to summary statistics, the data points behind means, medians and variance measures should be available. If there are restrictions on publicly sharing data—e.g. participant privacy or use of data from a third party—those must be specified.

Reviewer #1: Yes

**********

5. Is the manuscript presented in an intelligible fashion and written in standard English?

PLOS ONE does not copyedit accepted manuscripts, so the language in submitted articles must be clear, correct, and unambiguous. Any typographical or grammatical errors should be corrected at revision, so please note any specific errors here.

Reviewer #1: Yes

**********

6. Review Comments to the Author

Please use the space provided to explain your answers to the questions above. You may also include additional comments for the author, including concerns about dual publication, research ethics, or publication ethics. (Please upload your review as an attachment if it exceeds 20,000 characters)

Reviewer #1: Dear authors. I carefully went over the manuscript once more. I am pleased with the authors' efforts to revise the work. I believe that every query posed in the initial review has been addressed by the writers. The manuscript is now more comprehensive and easier to read. Its technical fit has improved in the interim. The manuscript as it stands, in my opinion, can advance knowledge. No further changes are required.

**********

7. PLOS authors have the option to publish the peer review history of their article (what does this mean?). If published, this will include your full peer review and any attached files.

If you choose “no”, your identity will remain anonymous but your review may still be made public.

**Do you want your identity to be public for this peer review?**  For information about this choice, including consent withdrawal, please see our Privacy Policy.

Reviewer #1: **Yes:** Gyanesh Kumar Tiwari

**********

# Editor report: Acceptance letter

**DOI:** 10.1371/journal.pone.0352778.r006

**Authors:** Vinícius Silva Belo

PONE-D-25-39709R2

PLOS One

Dear Dr. Hediger,

I'm pleased to inform you that your manuscript has been deemed suitable for publication in PLOS One. Congratulations! Your manuscript is now being handed over to our production team.

At this stage, our production department will prepare your paper for publication. This includes ensuring the following:

* All references, tables, and figures are properly cited

* All relevant supporting information is included in the manuscript submission,

* There are no issues that prevent the paper from being properly typeset

You will receive further instructions from the production team, including instructions on how to review your proof when it is ready. Please keep in mind that we are working through a large volume of accepted articles, so please give us a few days to review your paper and let you know the next and final steps.

Lastly, if your institution or institutions have a press office, please let them know about your upcoming paper now to help maximize its impact. If they'll be preparing press materials, please inform our press team within the next 48 hours. Your manuscript will remain under strict press embargo until 2 pm Eastern Time on the date of publication. For more information, please contact onepress@plos.org.

You will receive an invoice from PLOS for your publication fee after your manuscript has reached the completed accept phase. If you receive an email requesting payment before acceptance or for any other service, this may be a phishing scheme. Learn how to identify phishing emails and protect your accounts at https://explore.plos.org/phishing.

If we can help with anything else, please email us at customercare@plos.org.

Thank you for submitting your work to PLOS ONE and supporting open access.

Kind regards,

PLOS ONE Editorial Office Staff

on behalf of

Dr. Vinícius Silva Belo

Academic Editor

PLOS One
